# **β**3-Adrenergic receptor downregulation leads to adipocyte catecholamine resistance in obesity

**DOI:** 10.1172/JCI153357

**Published:** 2022-01-18

**Authors:** Joseph M. Valentine, Maryam Ahmadian, Omer Keinan, Mohammad Abu-Odeh, Peng Zhao, Xin Zhou, Mark P. Keller, Hui Gao, Ruth T. Yu, Christopher Liddle, Michael Downes, Jin Zhang, Aldons J. Lusis, Alan D. Attie, Ronald M. Evans, Mikael Rydén, Alan R. Saltiel

**Affiliations:** 1 Department of Medicine and; 2 Department of Pharmacology, Bioengineering, Chemistry, and Biochemistry, UCSD, San Diego, California, USA.; 3 Department of Biochemistry, University of Wisconsin-Madison, Madison, Wisconsin, USA.; 4 Department of Medicine (H7), Karolinska Institutet, Karolinska University Hospital, Huddinge, Stockholm, Sweden.; 5 Gene Expression Laboratory, Salk Institute for Biological Sciences, La Jolla, California, USA.; 6 Storr Liver Centre, Westmead Institute for Medical Research and Sydney School of Medicine, University of Sydney, Westmead, New South Wales, Australia.; 7 Department of Microbiology, Immunology, and Molecular Genetics, Department of Medicine, UCLA, Los Angeles, California, USA.

**Keywords:** Cell Biology, Metabolism, Adipose tissue, G proteins, Obesity

## Abstract

The dysregulation of energy homeostasis in obesity involves multihormone resistance. Although leptin and insulin resistance have been well characterized, catecholamine resistance remains largely unexplored. Murine **β**3-adrenergic receptor expression in adipocytes is orders of magnitude higher compared with that of other isoforms. While resistant to classical desensitization pathways, its mRNA (*Adrb3*) and protein expression are dramatically downregulated after ligand exposure (homologous desensitization). **β**3-Adrenergic receptor downregulation also occurs after high-fat diet feeding, concurrent with catecholamine resistance and elevated inflammation. This downregulation is recapitulated in vitro by TNF-**α** treatment (heterologous desensitization). Both homologous and heterologous desensitization of *Adrb3* were triggered by induction of the pseudokinase TRIB1 downstream of the EPAC/RAP2A/PI-PLC pathway. TRIB1 in turn degraded the primary transcriptional activator of *Adrb3*, CEBP**α**. EPAC/RAP inhibition enhanced catecholamine-stimulated lipolysis and energy expenditure in obese mice. Moreover, adipose tissue expression of genes in this pathway correlated with body weight extremes in a cohort of genetically diverse mice and with BMI in 2 independent cohorts of humans. These data implicate a signaling axis that may explain reduced hormone-stimulated lipolysis in obesity and resistance to therapeutic interventions with **β**3-adrenergic receptor agonists.

## Graphical abstract

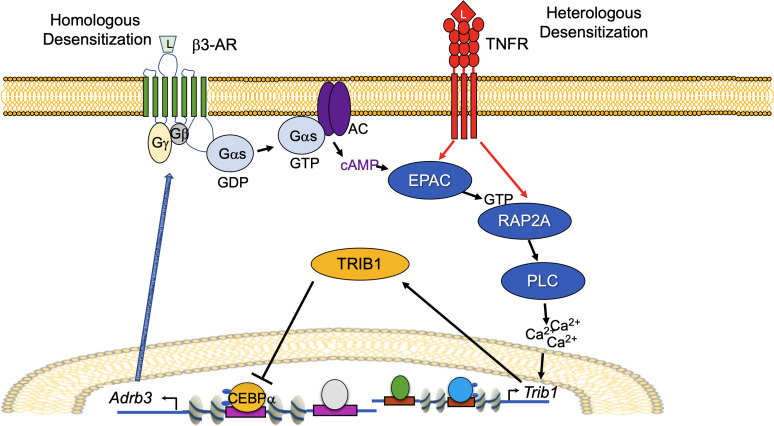

## Introduction

Body mass is governed by the balance between energy intake and expenditure, which are tightly coupled to ensure homeostasis even during periods of over- or undernutrition (1–4). Disruption of this balance leads to obesity, multihormone resistance, and metabolic inflexibility (5). While insulin and leptin resistance have been well characterized, catecholamine resistance remains relatively unexplored. Here, we define catecholamine resistance as the inability of adipocytes from obese individuals or animals to respond to catecholamines (synthetic or endogenous) to the same degree as those who are nonobese. Goldberg and Gordon first described this phenomenon in the early 1960s, observing that epinephrine-stimulated lipolysis was blunted in obese patients (6). Obesity-associated catecholamine resistance was confirmed and explored in more detail by Jensen (7–9), Arner (10, 11), and others in animal models (12–14) and humans (15–18). Catecholamine resistance is a key feature of the obese state and may even predict future weight gain in some patients (19, 20).

The rate-limiting step in adipocyte catecholamine signaling is activation of β-adrenergic receptors (β3-ARs) on the cell surface. While all 3 β-adrenergic subtypes are expressed in adipocytes, the β3-AR is by far the predominant form in mice, whereas expression patterns in humans are more variable (21, 22). The β1- and β2-ARs undergo classic β-arrestin–mediated desensitization within minutes of ligand exposure (23). In contrast, β3-AR is uniquely resistant to classical clathrin-meditated endocytosis (23) because it lacks a C-terminal β-arrestin–binding motif and the key phosphorylation sites responsible for internalization of β1- and β2-ARs (24). Furthermore, sustained β3-AR activation in the human myometrium does not result in desensitization during tocolytic therapy (25). Indeed, β3-AR desensitization appears to be context, organism, and cell-type specific (26). Ligand exposure decreases expression of β3-AR mRNA encoded by *Adrb3* (26–28). Adipocytes exposed to the inflammatory cytokine TNF-α also downregulate *Adrb3* (29).

Elevated adipose tissue inflammation and increased cytokine levels are well documented in obesity and type 2 diabetes mellitus (T2DM) (30–33) and may contribute to both insulin and catecholamine resistance (30, 34, 35). Adipose tissue inflammation is acutely catabolic and associated with increased lipolysis (36, 37). However, long-term high-fat diet–induced (HFD-induced) chronic inflammation is associated with repression of stimulated lipolysis and thermogenesis (38) as well as reduced expression of genes involved in lipolytic, fatty acid oxidation, and thermogenic pathways, including *Adrb3* (38–40).

The β3-AR is a G protein–coupled receptor functionally linked to Gαs and Gβγ subunits. Stimulation of β3-AR increases intracellular cAMP levels through activation of adenylyl cyclase. cAMP activates 2 major signaling cascades in adipocytes, the PKA and exchange protein directly activated by cAMP/Ras-related protein (EPAC/RAP) pathways. PKA catalyzes the phosphorylation of hormone-sensitive lipase (HSL) and perilipin to increase lipolysis (34, 41) and induces thermogenic gene transcription through activating transcription factor 2/cAMP-responsive element binding protein (ATF-2/CREB) transactivation (42, 43). While cAMP-dependent EPAC activation has received limited attention in adipocytes, it may be involved in regulation of leptin secretion (44, 45), adipocyte cell differentiation (46), and exocytosis (40). We report here that pathways controlling the negative regulation of *Adrb3* transcription by both homologous (β3-AR) and heterologous (inflammatory) signals converge at the EPAC/RAP2A/phosphoinositide-phospholipase C (PI-PLC) pathway, which orchestrates a cascade of transcriptional events to repress *Adrb3* gene expression through targeted degradation of CEBPα in adipocytes. This downregulation of *Adrb3* expression can be demonstrated in vitro in response to receptor activation or the inflammatory cytokine TNF-α and in vivo in response to HFD or agonist stimulation, resulting in catecholamine resistance. Inhibition of the EPAC/RAP pathway improved catecholamine sensitivity, as assessed by stimulated lipolysis and energy expenditure in obese mice. Furthermore, adipose tissue *ADRB3* and *RAP2A* expression correlates with both inflammatory gene expression and BMI in mouse strains as well as humans. Thus, this pathway may represent an axis for therapeutic intervention in obesity and related metabolic diseases, providing an approach to ensuring a durable response to β3-AR agonists in obesity treatment.

## Results

### Homologous desensitization of the adipocyte β3-AR produces catecholamine resistance in vitro and in vivo.

**Figure 1:**
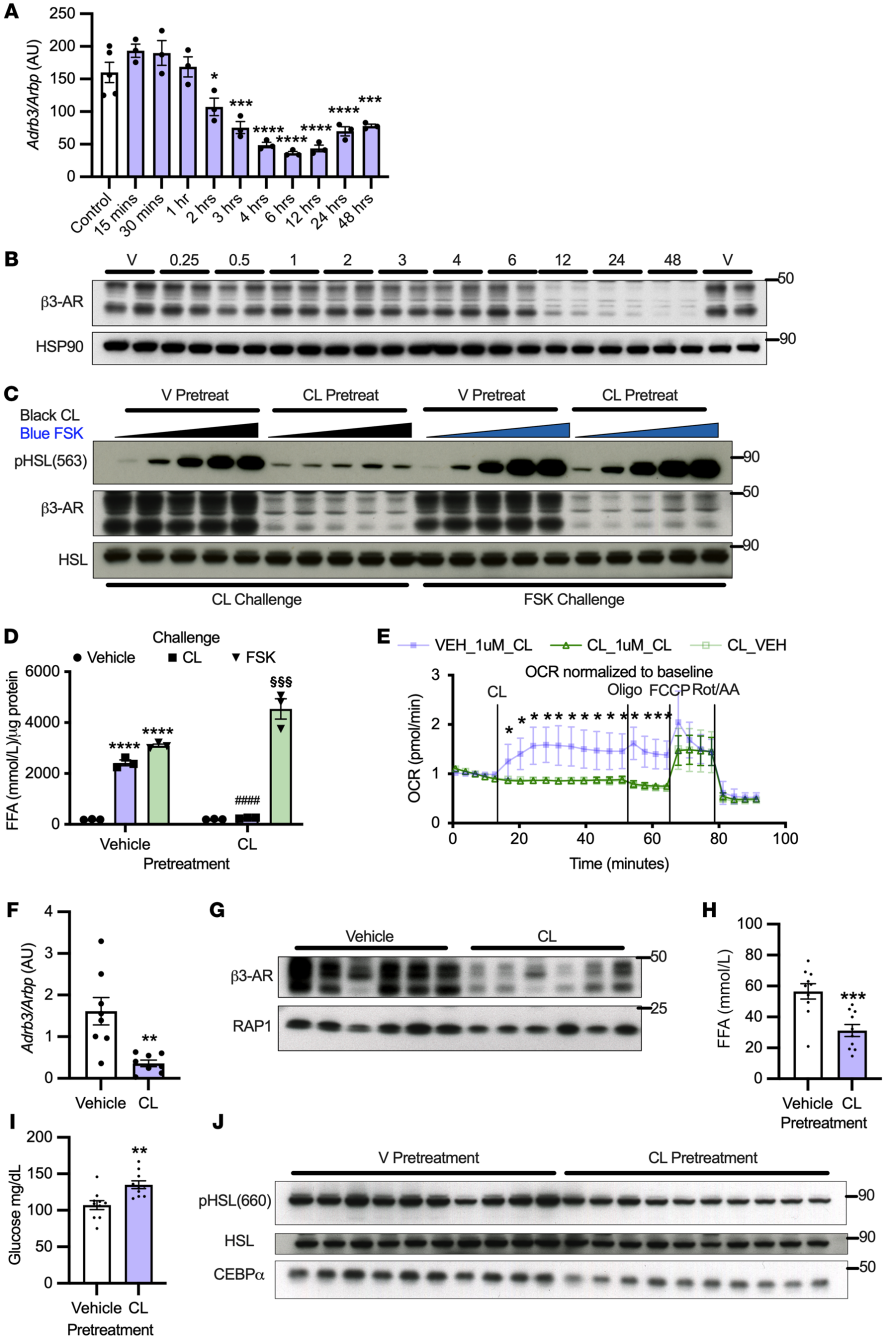
Homologous desensitization of the adipocyte β3-AR produces catecholamine resistance in vitro and in vivo. (**A**) *Adrb3* mRNA (*n* = 3 per group, repeated once) and (**B**) β3-AR were assessed in 3T3L1 adipocytes treated with 10 μM CL-316243 (*n* = 3 per group). (**C**) 3T3L1 adipocytes were pretreated for 48 hours with 10 μM CL-316243, then challenged with CL-316243 (1 μM–0.001 μM) or FSK (50 μM–0.05 μM) (representative *n* = 2 per group). (**D**) 3T3L1 adipocytes were pretreated for 48 hours with 10 μM CL-316243 and challenged with 1 μM CL-316243 or 50 μM FSK and FFA determined by absorbance (*n* = 3 per group). *Significance from vehicle-prevehicle challenge; ^#^significance from vehicle–pre–CL-316243 challenge to CL-316243–pre–CL-316243 challenge; ^§^significance from vehicle-pre-FSK challenge to CL-316243–pre–FSK challenge. (**E**) Oxygen consumption rate (OCR) after 18 hours pretreatment with 0.1 μM CL-316243 was determined by Seahorse in PPDIVs (*n* = 8 per group). *Significance from vehicle-prevehicle challenge in all groups. (**F** and **G**) *Adrb3* mRNA (*n* = 7–8 per group) and β3-AR protein expression (*n* = 6 per group) were determined in mouse iWAT 12 hours after 0.5 mg/kg CL-316243 i.p. injection. (**H** and **I**) Mice were pretreated with 0.5 mg/kg CL-316243 and challenged with 0.1 mg/kg CL-316243 12 hours later (*n* = 10 per group). (**H**) Serum FFA, (**I**) lipolysis-dependent glucose lowering, and (**J**) pHSL in iWAT were assessed. *Significance equal to that of control unless otherwise specified. One-way ANOVA with Dunnett’s multiple comparison (**A**); 2-way ANOVA with Tukey’s multiple comparisons (**D**); 2-way mixed ANOVA (time × treatment) with Tukey’s post hoc tests (**E**); independent sample *t* tests (**F**, **H**, and **I**). All error bars represent SEM. *P < 0.05; **P < 0.01; ***P < 0.001; ****P < 0.0001. ^####^*P* < 0.0001. ^§§§^*P* < 0.001.

We hypothesize that catecholamine resistance results from downregulation of β3-ARs in a cell-autonomous fashion. Therefore, we treated 3T3L1 adipocytes with the β3-AR selective agonist CL-316243 and determined *Adrb3* expression normalized to the housekeeping gene *Arbp*. *Adrb3* was significantly decreased within 2 hours of treatment and remained low across a 48-hour time span (Figure 1A). The decrease in mRNA preceded that of β3-AR protein expression (antibody and 10 μM CL-316243 dose validated for specificity using β3-AR–knockout adipocytes; Supplemental Figure 1A; supplemental material available online with this article; https://doi.org/10.1172/JCI153357DS1), which was dramatically downregulated 12 hours after CL-316243 treatment (Figure 1B). To understand whether loss of β3-AR results in homologous desensitization (reduced activation in response to ligand exposure), we pretreated adipocytes for 48 hours with CL-316243, which blocked phosphorylation of HSL, cAMP production, and free fatty acid (FFA)/glycerol release in response to a second 15-minute CL-316243 challenge, but did not alter the response to the adenylyl cyclase activator forskolin (FSK) (Figure 1, C and D, and Supplemental Figure 1, B and C). Together, these data suggest that the impairment in β-adrenergic–dependent lipolysis was due to a loss of the receptor and not downstream signaling. In addition, pretreatment of primary preadipocytes differentiated in vitro (PPDIVs) with 0.1 μM CL-316243 for 18 hours completely blocked CL-316243–dependent (1 μM) respiration without altering maximum respiration (Figure 1E). Consistent with our in vitro data, *Adrb3* mRNA and protein were downregulated in adipose tissue (inguinal white adipose tissue [iWAT]) from mice treated with CL-316243 for 12 hours (Figure 1, F and G). Furthermore, mice pretreated with CL-316243 for 12 hours exhibited impaired FFA secretion, HSL phosphorylation in iWAT, and catecholamine-dependent lowering of blood glucose (as described previously, ref. 47) in response to a second CL-316243 challenge (Figure 1, H–J).

### Homologous desensitization is specific for the β3-AR and cAMP dependent.

To determine whether *Adrb3* downregulation is generalizable to alternative intracellular signals that increase cAMP, we demonstrated that pretreatment with 0.5 to 5 μM FSK downregulated *Adrb3* mRNA in 3T3L1 adipocytes (Supplemental Figure 1D). FSK pretreatment for 48 hours also downregulated β3-AR protein expression, which led to impaired catecholamine signaling, as measured by phosphorylation of P38 in response to increasing concentrations of CL-316243 (Supplemental Figure 1E). We next tested to determine whether increasing cAMP with a β1-AR– (dobutamine) or β2-AR–specific agonist (formoterol) could also downregulate *Adrb3*. Formoterol and dobutamine treatment downregulated *Adrb3* in a dose- and time-dependent fashion while upregulating the cAMP-responsive genes *Il6* and *Nr4a3* as well as *Adrb1* and *Adrb2* expression (Supplemental Figure 2, A–T). Thus, activation of β1- and β2-ARs does not produce desensitization to ligand exposure through downregulation of their own mRNA, but may modestly increase them to compensate for Adrb3 downregulation.

### Adrb3 desensitization depends on EPAC but not PKA signaling.

**Figure 2:**
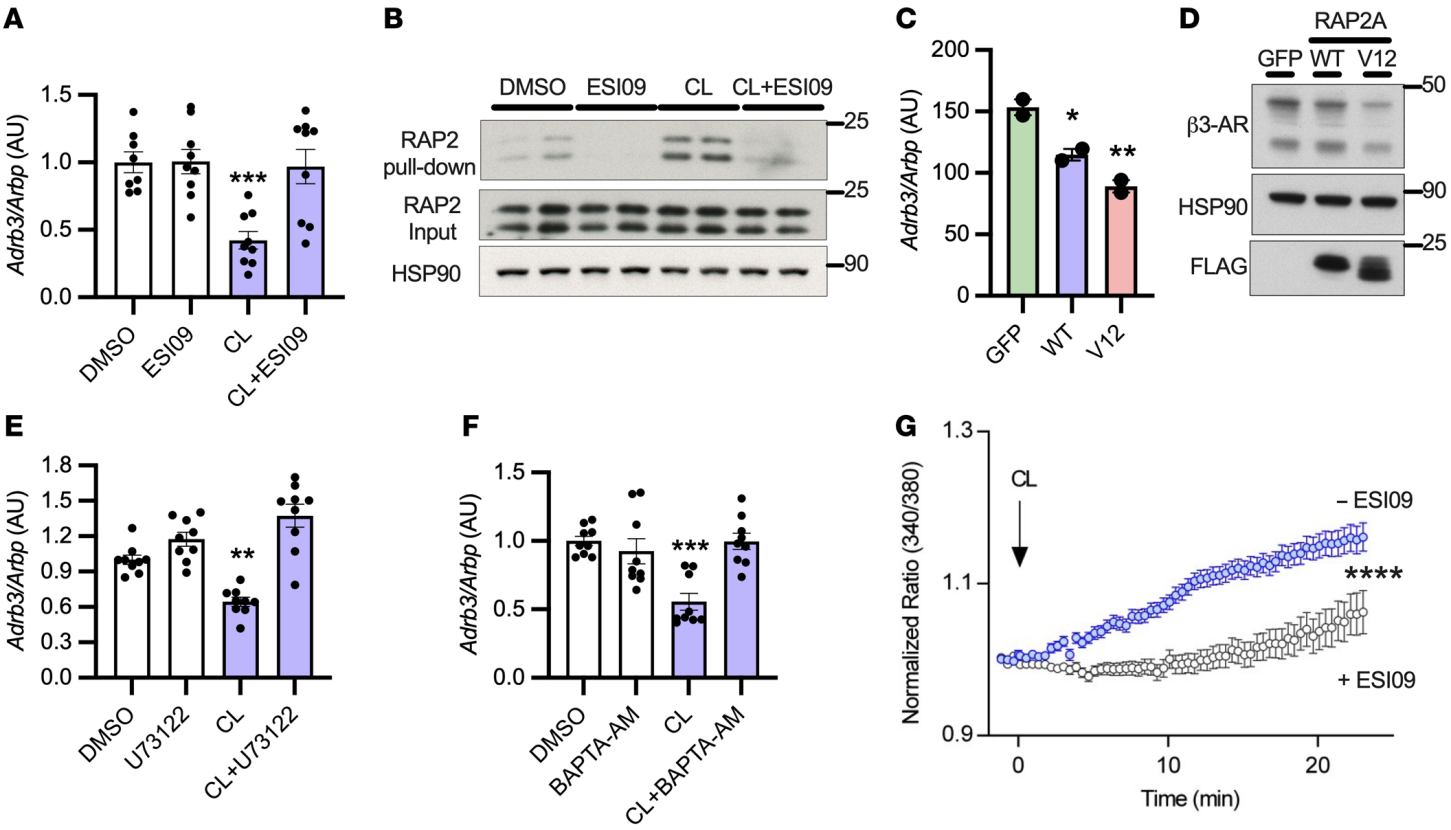
Desensitization of β3-AR depends on EPAC/RAP2A/PLC pathway activation. (**A**) 3T3L1 adipocytes were pretreated with 10 μM ESI09 for 1 hour, then challenged with 10 μM CL-316243 for 3 hours (*n* = 3 per group, 3 independent experiments). (**B**) Active (GTP bound) RAP2 was determined by pulldown followed by Western blotting with RAP2 antibody (*n* = 2 per group). (**C** and **D**) FLAG-tagged RAP2A WT or constitutively active RAP2A (V12) was electroporated into 3T3L1 adipocytes (*n* = 1–2 per group, repeated once). (**E** and **F**) 3T3L1 adipocytes were pretreated for 1 hour with 10 μM U73122 or 50 μM BAPTA-AM, followed by 3 hours challenge with 10 μM CL-316243 (*n* = 3 per group, 3 independent experiments). (**G**) 3T3L1 adipocytes were pretreated with 10 μM ESI09 for 1 hour, challenged with 10 μM CL-316243, and calcium flux assessed in live cells using Fura2-AM (91 randomly chosen cells from 4 experiments [blue] and 101 from 3 experiments [gray]). Graph represents the subpopulation of cells that responded to CL-316243 (~20%). *Significance compared with control or GFP in all experiments. One-way ANOVA with Tukey’s post hoc comparisons (**A**, **C**, **E**, and **F**); independent samples *t* test (**G**). All error bars represent SEM. *P < 0.05; **P < 0.01; ***P < 0.001; ****P < 0.0001.

β3-AR stimulation activates PKA to modulate gene transcription through ATFs and CREB (42, 43). Therefore, we pretreated 3T3L1 adipocytes with the PKA inhibitor H89 prior to CL-316243 challenge, which did not prevent the downregulation of *Adrb3* in response to CL-316243, but did inhibit the phosphorylation of HSL (Supplemental Figure 3, A–C). cAMP also activates the small G protein RAP by binding to and activating its cognate guanyl nucleotide exchange factor (GEF) EPAC (48, 49). Pretreatment of adipocytes with the EPAC inhibitor ESI-09 completely prevented the downregulation of *Adrb3* in response to CL-316243 (Figure 2A). Importantly, CL-316243 and FSK activated RAP2, but not RAP1, proteins in an EPAC-dependent manner (Figure 2B and Supplemental Figure 3, D and E). Overexpression of RAP2A (WT) or a constitutively active RAP2A (V12) mutant in adipocytes downregulated *Adrb3* mRNA and β3-AR protein (Figure 2, C and D). Together, these data indicate that cAMP-dependent EPAC/RAP2A pathway activation specifically mediates homologous desensitization of β3-AR in adipocytes.

### Activation of PI-PLC is required for Adrb3 downregulation.

RAP2A can activate PLCε (50), and pretreatment of adipocytes with the pan PLC inhibitor U73122 completely blocked the downregulation of *Adrb3* in response to CL-316243 (Figure 2E). Upon its activation, PI-PLC mobilizes calcium to the cytoplasm, and pretreatment of adipocytes with the cell-permeable calcium chelator BAPTA-AM prevented the downregulation of *Adrb3* in response to CL-316243 (Figure 2F). Conversely, the calcium ionophore A23187 or ionomycin downregulated *Adrb3* (Supplemental Figure 4, A and B). Live-cell imaging of 3T3L1 adipocytes revealed that CL-316243 treatment increased calcium mobilization in a subpopulation of cells, which could be blocked by pretreatment with ESI-09 (Figure 2G), indicating that EPAC/RAP2A activation stimulates PI-PLC to increase cytosolic calcium in adipocytes, leading to downregulation of *Adrb3*.

### Heterologous desensitization of Adrb3 occurs by inflammatory cytokine stimulation in vitro and in obese mice with catecholamine resistance.

**Figure 3:**
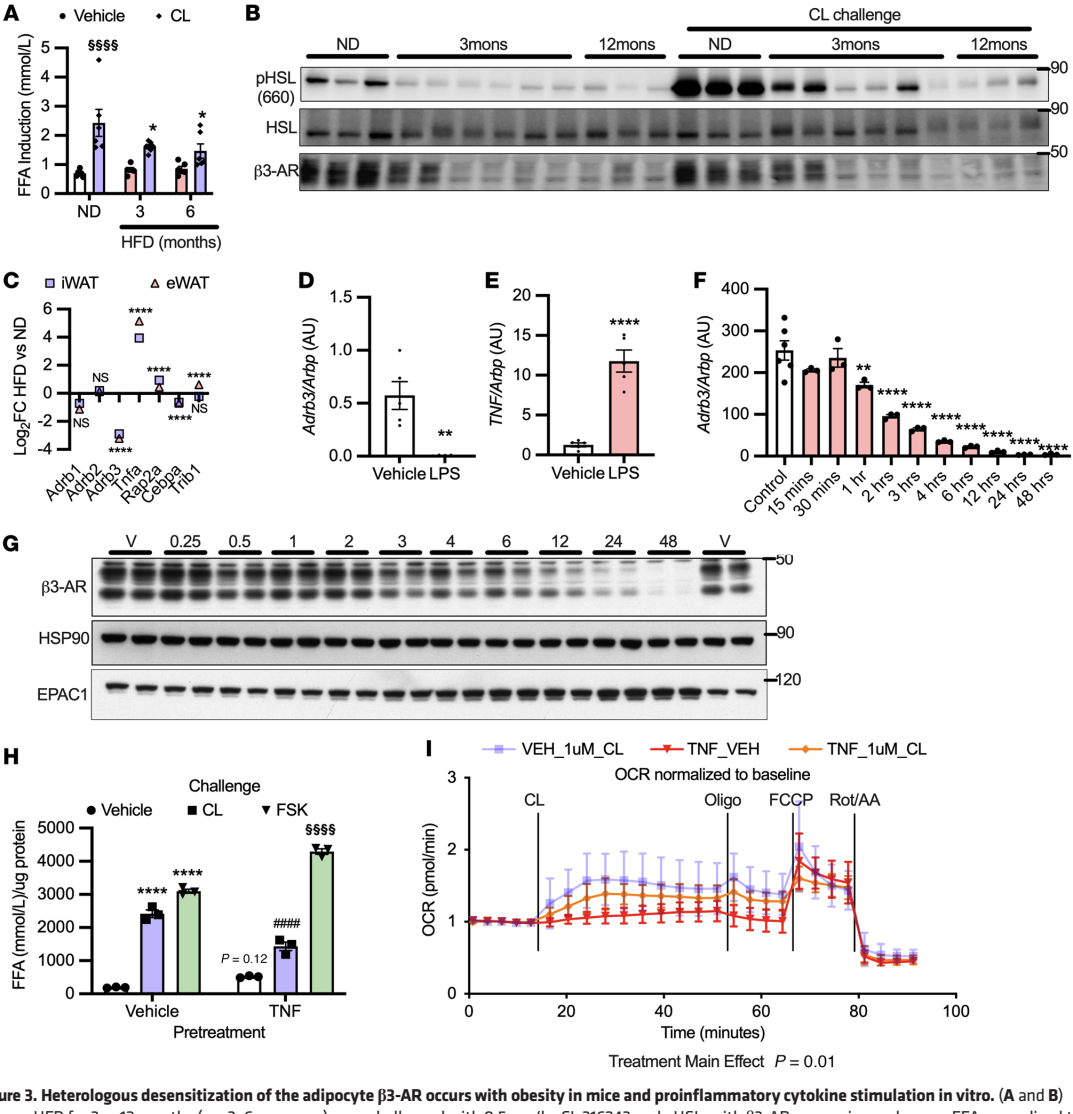
Heterologous desensitization of the adipocyte β3-AR occurs with obesity in mice and proinflammatory cytokine stimulation in vitro. (**A** and **B**) Mice on HFD for 3 or 12 months (*n* = 3–6 per group) were challenged with 0.5 mg/kg CL-316243 and pHSL, with β3-AR expression and serum FFA normalized to baseline measured as before. ^§^Significance compared with vehicle; *significance compared with ND-CL-316243. (**C**) RNA-Seq from isolated adipocytes of iWAT and eWAT in mice fed HFD for 16 weeks (*n* = 3 per group). *Significance between HFD and ND for the listed genes. (**D** and **E**) Mice were treated with 10 mg/kg LPS for 18 hours and gene expression assessed in eWAT (*n* = 5 per group). (**F**) *Adrb3* (*n* = 3 per group repeated once) and (**G**) β3-AR were assessed as before with 17 ng/mL TNF-α treatment. *n* = 3 per group. (**H**) 3T3L1 adipocytes were pretreated and challenged as before then FFAs (*n* = 3 per group) measured as in Figure 1, D and E. (**H**) Experiments shown in part H had the same experimental design as in Figure 1D, except pretreatment was 17 ng/mL TNF-α. Significance symbols are as described for Figure 1, D and E. (**I**) Oxygen consumption rates after 18 hours pretreatment with 17 ng/mL TNF-α were determined by Seahorse using PPDIVs (*n* = 8 per group). *Significance compared with control unless otherwise specified. Controls in **I** were also used for Figure 1 for cognate experiments, as these assays were carried out simultaneously. Two-way ANOVA with Šidák’s (**A**) and Tukey’s (**H**) multiple comparisons; *t* tests and FDR applied to correct for multiple comparisons (**C**); independent samples *t* test (**D** and **E**); 1-way ANOVA with Dunnett’s post hoc test (**F**); 2-way mixed ANOVA (time × treatment) with Tukey’s post hoc test (**I**). Error bars represent SEM. *P < 0.05; **P < 0.01; ***P < 0.001; ****P < 0.0001. ^####^*P* < 0.0001. ^§§§§^*P* < 0.001.

Mice fed a HFD for 3 or 12 months exhibited catecholamine resistance, characterized by reduced CL-316243–dependent rise in serum FFA, phosphorylation of HSL in WAT, and reduced β3-AR protein expression (Figure 3, A and B, and Supplemental Figure 4C). Therefore, we conducted RNA-Seq on isolated adipocytes from epididymal and subcutaneous-inguinal adipose depots derived from mice fed a normal diet (ND) or HFD for 4 months. *Adrb3* expression was several hundred-fold higher than *Adrb1* or *Adrb2* in adipocytes from both depots of mice on ND (Supplemental Figure 4D). Furthermore, *Adrb3* expression was markedly downregulated after HFD feeding, while *Adrb1* and *Adrb2* remained unchanged (Figure 3C). *Rap2a* and the inflammatory cytokine *Tnf* were concurrently upregulated in adipocytes from HFD-fed mice (Figure 3C). In an orthogonal approach to determining whether inflammation downregulates *Adrb3* in vivo, we treated mice with LPS, which increased *Tnf* and downregulated *Adrb3* expression in epididymal WAT (eWAT) (Figure 3, D and E).

### TNF-α–induced heterologous desensitization of β3-AR impairs β-adrenergic–stimulated lipolysis.

Because TNF was elevated after HFD or LPS treatment and it is reported to increase cAMP through inhibition of PDEs (36, 51), we treated 3T3L1 adipocytes with TNF-α, which decreased *Adrb3* expression with temporal kinetics similar to those of CL-316243 (Figure 3F). Again, *Adrb3* mRNA decreased prior to downregulation of β3-AR protein expression (Figure 3G). To determine whether decreased β3-AR expression after TNF-α treatment results in desensitization to agonist, we pretreated adipocytes with TNF-α for 48 hours, which dramatically reduced CL-316243–stimulated cAMP production and had modest effects on FSK-stimulated cAMP production (Supplemental Figure 4E). The effect of TNF-α pretreatment on FSK-stimulated cAMP production may result from alterations to expression/activity of PDE3B (34). In contrast, CL-316243–stimulated lipolysis was reduced by TNF-α pretreatment, while FSK-stimulated lipolysis was enhanced (Figure 3H and Supplemental Figure 4F). Furthermore, TNF-α pretreatment had a modest effect on CL-316243–stimulated respiration in PPDIVs (Figure 3I). Collectively, these data indicate that the primary defect by which TNF-α reduces catecholamine sensitivity is through downregulation of β3-AR.

### Both homologous and heterologous desensitization of β3-AR utilize the same signaling network.

**Figure 4:**
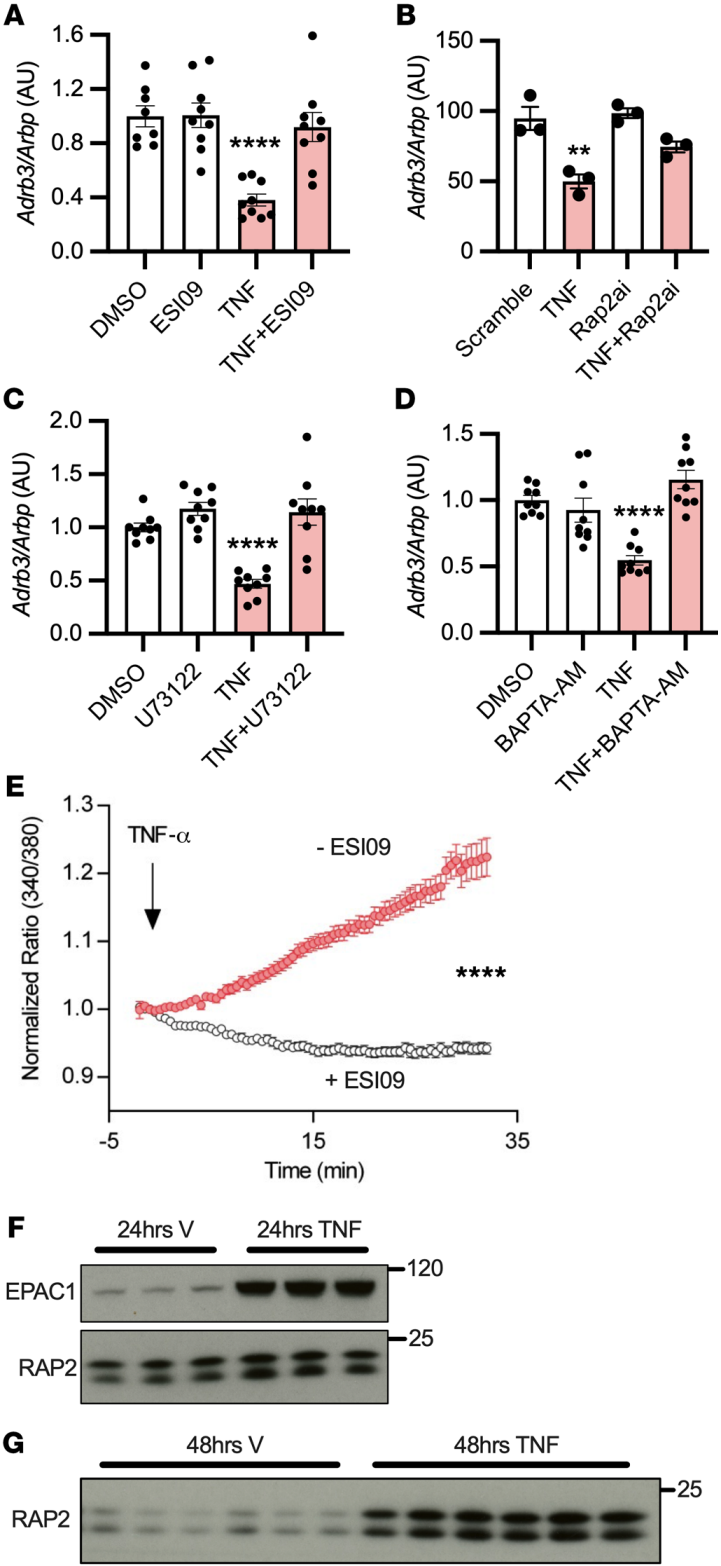
Heterologous desensitization of β3-AR occurs through activation of the same EPAC/RAP2A/PLC pathway. (**A**) 3T3L1 adipocytes were pretreated with 10 μM ESI09 for 1 hour, then challenged with 17 ng/mL TNF-α for 3 hours (*n* = 3 per group from 3 independent experiments). (**B**) 3T3L1 adipocytes were transfected with siRNA against RAP2A and challenged with 17 ng/mL TNF-α (3 hours) (*n* = 3 per group). (**C** and **D**) 3T3L1 adipocytes were pretreated for 1 hour with 10 μM U73122 or 50 μM BAPTA-AM, followed by 3 hours challenge with 17 ng/mL TNF-α (*n* = 3 per group from 3 independent experiments). (**E**) 3T3L1 adipocytes were pretreated with vehicle or 10 μM ESI09 for 1 hour and challenged with 17 ng/mL TNF-α; calcium flux was assessed in live cells using Fura2-AM (106 randomly chosen cells from 4 experiments [red] and 86 from 3 experiments [gray]). Graph represents the subpopulation of cells that responded to TNF-α (~20%). (**F** and **G**) 3T3L1 adipocytes were treated for the indicated times with 17 ng/mL TNF-α (*n* = 3–6 per group). Data points for DMSO control and inhibitor alone in **A**, **C**, and **D** were also used for Figure 2 for their cognate experiments, as these assays were carried out simultaneously. *Significance compared with control or GFP unless otherwise specified. One-way ANOVA with Tukey’s post hoc comparisons (**A**–**D**); independent samples *t* test (**E**). Error bars represent SEM. **P < 0.01; ****P < 0.0001.

Similarly to observations with CL-316243, EPAC inhibition prevented the downregulation of *Adrb3* after TNF-α treatment (Figure 4A), whereas H89 had no effect (Supplemental Figure 4G). Additionally, RAP2A knockdown blocked TNF-α–dependent *Adrb3* downregulation (Figure 4B). Pretreatment of adipocytes with either the PLC inhibitor (U73122) or calcium chelator (BAPTA-AM) prevented the downregulation of *Adrb3* in response to TNF-α (Figure 4, C and D). TNF-α treatment again increased EPAC/RAP-dependent calcium mobilization in a subpopulation of adipocytes (Figure 4E). Interestingly, TNF-α produced a more robust repression of *Adrb3* (Figure 3F), which may have resulted from the induction of both EPAC and RAP2 proteins after TNF-α treatment (Figure 3G and Figure 4, F and G). Taken together, these data indicate that both homologous and heterologous desensitization occur through an EPAC/RAP2A/PI-PLC signaling cascade, leading to calcium-dependent transcriptional repression of *Adrb3*.

### A synthesized transcriptional repressor is required for homologous and heterologous desensitization.

Elevated cytosolic calcium or cAMP induces genes/proteins for immediate responses to extracellular signals. Therefore, we hypothesized that a synthesized transcription repressor was required for *Adrb3* downregulation. Pretreatment of 3T3L1 adipocytes with the protein synthesis inhibitor cycloheximide prevented the downregulation of *Adrb3* in response to both TNF-α and CL-316243 (Supplemental Figure 5A), indicating that a synthesized transcriptional repressor of *Adrb3* is obligatory for homologous and heterologous desensitization.

### The pseudokinase Trib1 is induced and required for Adrb3 downregulation.

**Figure 5:**
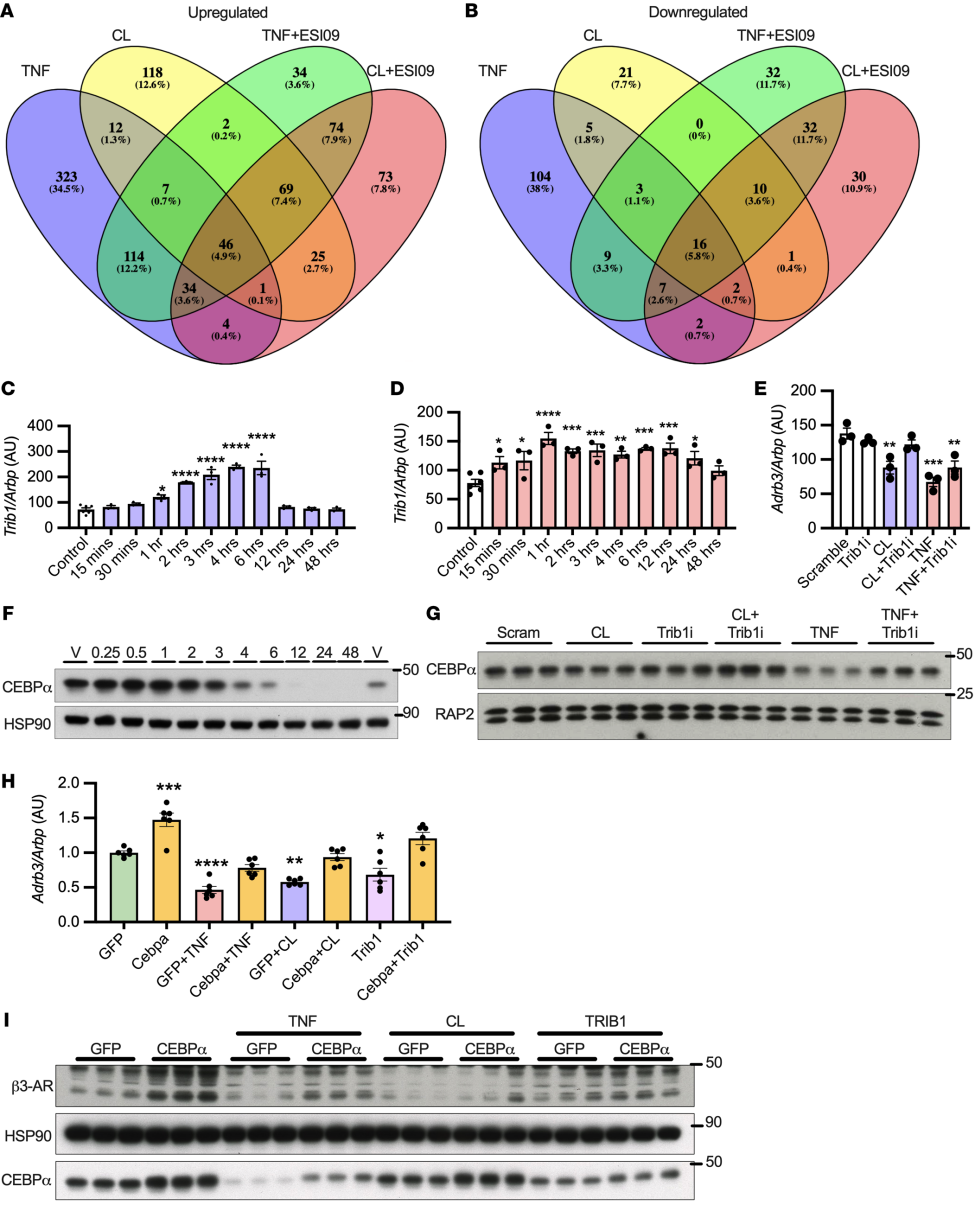
Pseudokinase Trib1-mediated degradation of Cebpa is induced and required for Adrb3 downregulation. (**A** and **B**) RNASeq was conducted on 3T3L1 adipocytes treated with 17 ng/mL TNF-α or 10 μM CL-316243 for 3 hours with or without 10 μM ESI09 (*n* = 3 per group, 1 sample from 3 independent experiments). (**C** and **D**) 3T3L1 adipocytes were treated with 10 μM CL-316243 or 17 ng/mL TNF-α (*n* = 3 per group). (**E**) 3T3L1 adipocytes were treated with *Trib1* siRNA, then challenged with 1 μM CL-316243 or 17 ng/mL TNF-α. (**F**) 3T3L1 adipocytes treated with 17 ng/mL TNF-α for up to 48 hours (representative of *n* = 3 replicates). (**G**) 3T3L1 adipocytes were treated with siRNA against *Trib1*, then challenged with 1 μM CL-316243 or 17 ng/mL TNF-α for 3 hours (*n* = 3 per group). (**H** and **I**) *Cebp*α and *Trib1* overexpression in 3T3L1 adipocytes was achieved by lentivirus followed by treatment with 1 μM CL-316243 or 17 ng/mL TNF-α for (**H**) 3 hours (*n* = 6 per group, from 2 independent experiments) or (**I**) 18 hours (*n* = 3 per group repeated once with similar results). *Significance compared with control or GFP unless otherwise specified. *t* test and FDRs applied to correct for multiple comparisons(**A** and **B**); 1-way ANOVA with Dunnett’s (**C**–**E**) or Tukey’s post hoc test (**H**). Error bars represent SEM. *P < 0.05; **P < 0.01; ***P < 0.001; ****P < 0.0001.

We conducted RNA-Seq on 3T3L1 adipocytes treated with TNF-α or CL-316243 with or without ESI-09. Twelve genes were induced and 5 repressed by both TNF-α and CL-316243 in a manner sensitive to EPAC inhibition (Figure 5, A and B). While *Adrb3* was 1 of the 5 repressed genes, *Adrb1* and *Adrb2* were not altered or modestly upregulated in response to TNF or CL-316243 with or without ESI-09 (Supplemental Figure 5, B and C). Interestingly, 1 of the 12 genes (*Trib1*) was induced by TNF-α and CL-316243 in an EPAC-dependent manner prior to *Adrb3* downregulation (Figure 5, C and D). The calcium ionophore A23187 or ionomycin also induced *Trib1* expression (Supplemental Figure 6, A and B) and subsequently downregulated *Adrb3* (Supplemental Figure 4, A and B). Knockdown of *Trib1* in 3T3L1 adipocytes prevented the downregulation of *Adrb3* in response to CL-316243 and partially blocked the downregulation from TNF-α treatment (Figure 5E), suggesting that TRIB1 induction is required for transcriptional regulation of *Adrb3*.

### TRIB1 desensitizes β3-AR by targeting its transactivator CEBPα.

TRIB1 can remove CEBPα from the nucleus and recruit COP-1 to degrade it (52). Furthermore, CEBPα is the primary transactivator for *Adrb3* (53); therefore, we focused on this transcription factor. CEBPα expression decreased over time with TNF-α (Figure 5F) and in vivo with HFD feeding (Figure 3C) or 12 hours of CL-316243 treatment (Figure 1J). Three hours of TNF-α treatment decreased CEBPα protein, which could be rescued by *Trib1* knockdown (Figure 5G). The effect of CL-316243 on CEBPα protein was modest at this time point; however, *Trib1* knockdown in combination with CL-316243 increased CEBPα protein expression (Figure 5G). Knockdown of *Trib1* also blocked the downregulation of β3-AR and degradation of CEBPα in response to 18 hours of CL-316243 treatment (Supplemental Figure 6C). Overexpression of constitutively active V12 or WT RAP2A using lentivirus in PPDIVs decreased CEBPα and subsequently β3-AR protein expression (Supplemental Figure 6D). CEBPα and PPARγ reciprocally regulate each other’s expression to enhance adipocyte differentiation; however, *Pparg* gene expression was only modestly, yet nonsignificantly, decreased after TNF-α treatment, with no change in its target *Fabp4* (Supplemental Figure 6, E and F), suggesting that the changes to CEBPα expression in response to TNF-α or CL-316243 do not alter its normal function in adipocyte differentiation.

To further support the hypothesis that CEBPα and TRIB1 regulate the expression of *Adrb3*/β3-AR in homologous and heterologous desensitization, we overexpressed them in 3T3L1 adipocytes using lentivirus. CEBPα overexpression increased *Adrb3*/β3-AR and also prevented their downregulation in response to TNF-α or CL-316243 treatment (Figure 5, H and I). Conversely, TRIB1 overexpression downregulated *Adrb3*/β3-AR, which was blocked by cooverexpression of CEBPα (Figure 5, H and I). Taken together, these data indicate that both homologous and heterologous desensitization of β3-AR converge through activation of EPAC/RAP2A/PI-PLC–dependent Ca^2+^ stimulated induction of *Trib1*, which in turn leads to the degradation of CEBPα and downregulation of *Adrb3*/β3-AR.

### Inhibition of EPAC/RAP signaling rescues catecholamine resistance, increases energy expenditure, and subsequently causes weight loss in obese mice.

**Figure 6:**
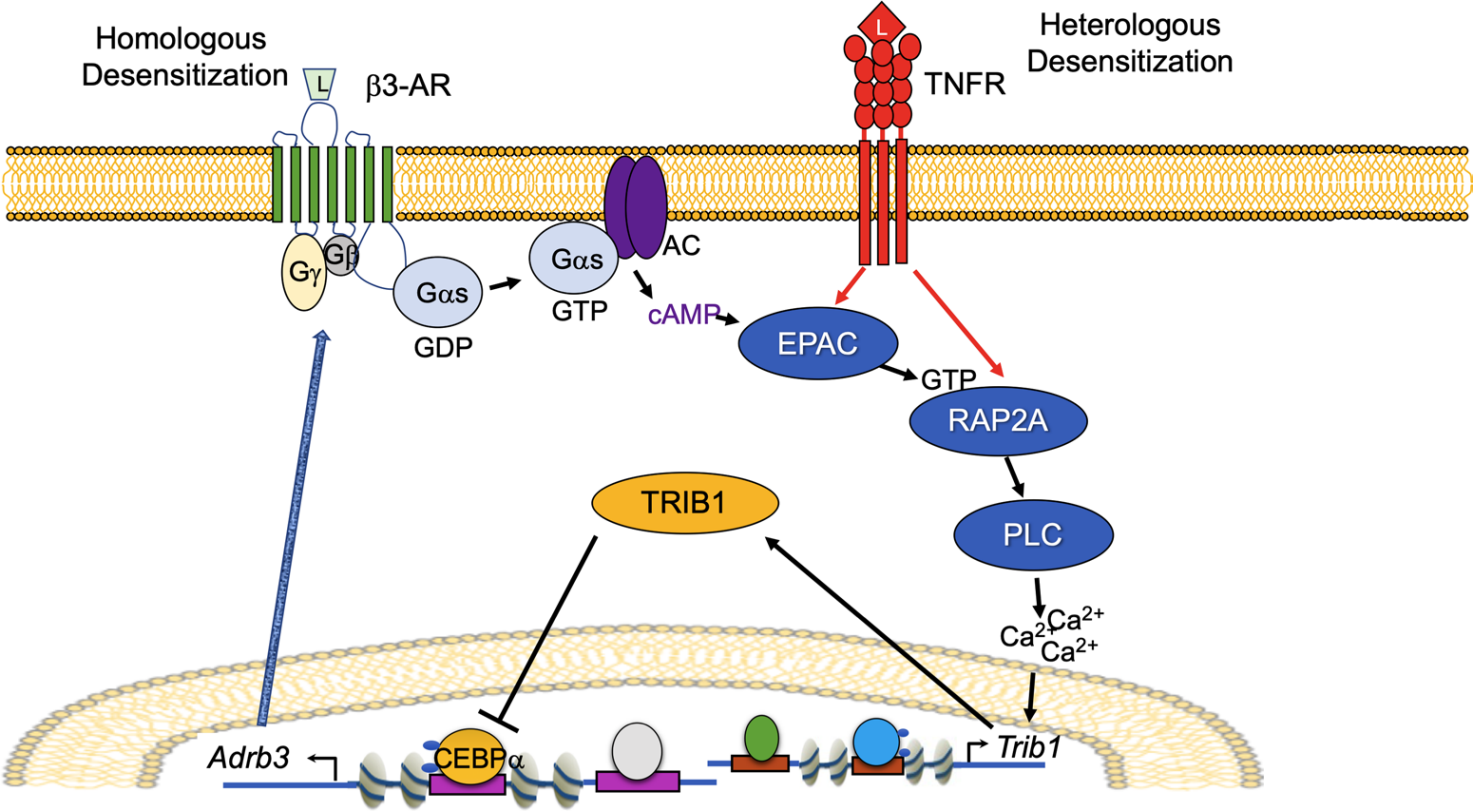
Model of signaling events leading to homologous and heterologous desensitization in adipocytes. Both homologous (β3-AR activation dependent) and heterologous (TNF-α dependent) desensitization of β3-AR converge through activation of EPAC/RAP2A/PI-PLC–dependent Ca^2+^–stimulated induction of *Trib1*, which in turn leads to the degradation of CEBPα and downregulation of *Adrb3*/β3-AR.

**Figure 7:**
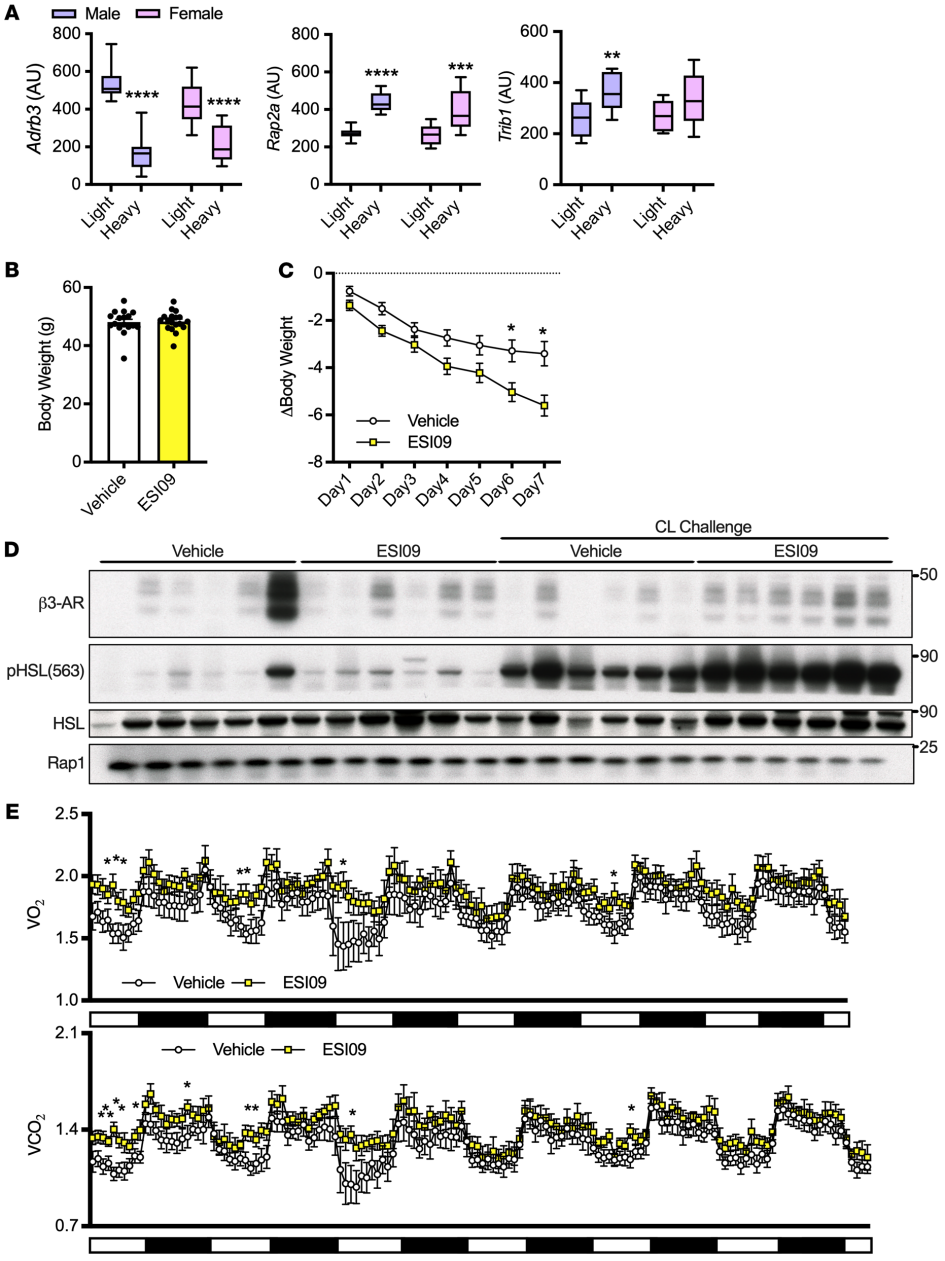
Inflammation drives EPAC/RAP-dependent catecholamine resistance in obesity. (**A**) eWAT of male and female mice with highest and lowest body weights (*n* = 10 per group, body weights in Supplemental Figure 7) in diversity outbred mice (*n* = 10 per group). (**B**) Starting body weights for mice fed HFD for 18 weeks before ESI-09 or vehicle treatment. (**C**) Body weight change in these mice during ESI-09 (10 mg/kg) or vehicle treatment (*n* = 17–18 per group). (**D**) β3-AR and pHSL from eWAT of mice in **C**; 24 hours after ESI09 treatment cessation, mice were challenged with 0.1 mg/kg CL-316243 or saline for 20 minutes (*n* = 6 per group). (**E**) Whole body oxygen consumption (VO_2_) and carbon dioxide production (VCO_2_) measured in a second cohort of mice fed HFD for 16 weeks with or without ESI-09 (10 mg/kg) for 1 week (*n* = 7–8 per group). Two-way ANOVA and Šidák’s post hoc comparison(**A**); independent samples *t* test (**B**); 2-way mixed ANOVA (day repeated measure and drug independent) with Šidák’s post hoc comparisons (**C**); 2-way mixed model (drug × time) and Fisher’s LSD post hoc test (**E**). *Significance compared with control unless otherwise specified. Error bars represent SEM. *P < 0.05; **P < 0.01; ***P < 0.001; ****P < 0.0001.

We assessed the generalizability of our model for homologous and heterologous desensitization (Figure 6) by utilizing a cohort of diversity outbred mice derived from intercrossing 8 different founder lines with varying body weights and metabolic traits, as previously described (54, 55). Consistent with our in vitro data, *Adrb3* expression was significantly lower, while *Rap2a* expression was elevated in eWAT from heavier compared with lighter mice of both sexes (Figure 7A). Body weights for these mice are presented in Supplemental Figure 7A. *Trib1* expression was also significantly higher in heavier male mice and trended toward elevated in female mice (Figure 7A). We then fed C57BL/6J mice HFD for 18 weeks and treated them once daily for 7 days with the EPAC/RAP inhibitor ESI-09 (10 mg/kg); body weights did not differ at the commencement of treatment (Figure 7B). Mice treated with ESI-09 lost significantly more weight than those treated with vehicles (Figure 7C). ESI-09 treatment also enhanced catecholamine signaling, demonstrated by higher phosphorylation of HSL in eWAT after 0.1 mg/kg CL-316243 challenge compared with vehicle-treated CL-316243–challenged obese mice (Figure 7D) with equal loading demonstrated by RAP1. iWAT from ESI-09–treated mice showed similar although less striking effects on pHSL (Supplemental Figure 7B). Furthermore, β3-AR protein expression in eWAT and iWAT were increased by ESI-09 treatment (Figure 7D and Supplemental Figure 7B). To determine the mechanism of weight loss in these mice, we put a second cohort on HFD for 16 weeks and conducted metabolic cage studies during the ESI-09 injection week. Again, body weights were not different at the commencement of treatment, and ESI-09 resulted in greater weight loss (Supplemental Figure 7, C and D). ESI-09 treatment also enhanced whole-body oxygen consumption, CO_2_ production, and energy expenditure (Figure 7E and Supplemental Figure 7E) without altering food consumption, respiratory exchange ratio, or activity (Supplemental Figure 7, E and F). Mice fed HFD for 16 to 18 weeks had elevated inflammatory gene transcription and crown-like structures, which were unaffected by EPAC inhibition (Supplemental Figure 7G and Supplemental Figure 8A). These results are consistent with our hypothesis that inflammation is upstream of EPAC/RAP2A activation/induction. We also saw no difference in browning gene transcription or UCP1 staining of iWAT in HFD-fed mice treated with ESI-09 or vehicle (Supplemental Figure 8, B–D). Collectively, downregulation of β3-AR protein drives catecholamine resistance in HFD-fed mice, which can be rescued by EPAC/RAP inhibition, resulting in enhanced lipolysis, energy expenditure, and subsequently, weight loss.

### Adipose tissue expression of ADRB3, RAP2A, and TRIB1 correlates with inflammatory marker and anthropometric measurements in 2 cohorts of humans.

**Figure 8:**
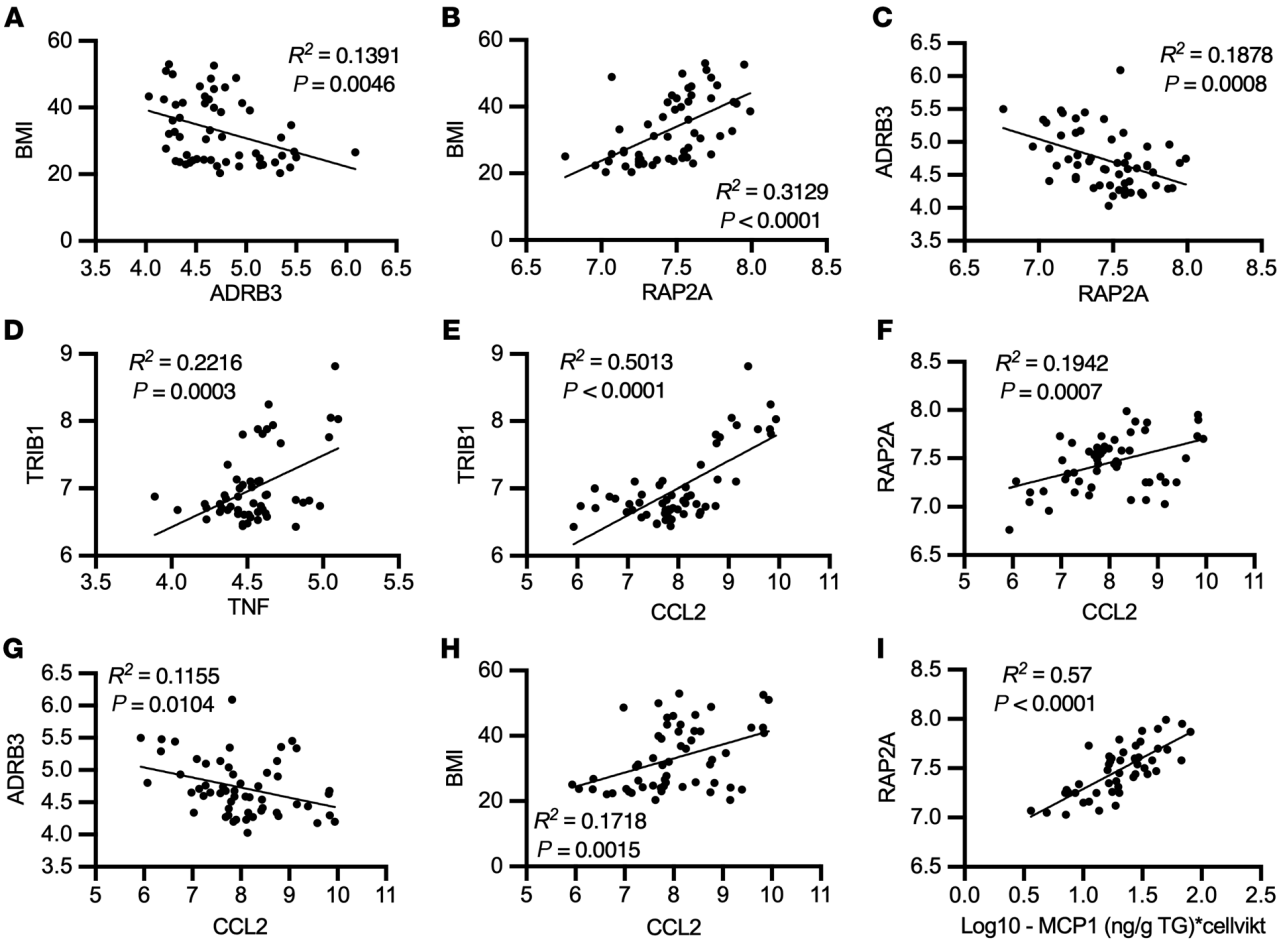
Human subcutaneous abdominal adipose tissue gene expression data of pathway members shown in Figure 6 correlate with BMI, inflammation, and each other. (**A**–**H**) Correlations between gene expression levels from microarray of human abdominal subcutaneous adipose tissue from women with varying BMIs (*n* = 56). (**I**) Correlations between isolated adipocyte secretion of MCP-1 and gene expression from these participants. Linear regression (**A**–**I**).

To determine the relevance of EPAC/RAP2A signaling in human obesity, we performed linear regression analysis on microarray data from abdominal subcutaneous adipose tissue of 56 women with or without obesity. *ADRB3* expression was negatively correlated with BMI (Figure 8A). In contrast, *RAP2A* expression was positively correlated with BMI (Figure 8B) and negatively correlated with *ADRB3* expression (Figure 8C). Consistent with our in vitro studies, *TNF* correlated positively with *TRIB1* expression (Figure 8D). The primary readout for TNF-α signaling, CCL2 expression, also correlated positively with *TRIB1*, *RAP2A*, and BMI and negatively with *ADRB3* (Figure 8, E–H). Furthermore, isolated adipocyte MCP-1 (the protein product of CCL2) secretion levels correlated inversely with *ADRB3* and positively with *RAP2A* in this cohort (Figure 8I and Supplemental Figure 9A). The correlations between *RAP2A*-BMI and *RAP2A*-CCL2, as well as between *CCL2*-ADRB3 and *CCL2*-BMI were confirmed in abdominal subcutaneous adipose tissue from 770 men with a broad range of BMIs (Supplemental Figure 9, B–E). Regression analysis revealed RAP2A as a predictor of *ADRB1* or *ADRB2* and *ADRB1* or *ADRB2* as predictors of BMI, although these associations were weaker than those observed with *ADRB3* (Supplemental Figure 9, F–I) in this data set. Inclusion of *TNF*, *TRIB1*, *ADRB3*, *RAP2A*, and *CCL2* into a multiple regression analysis demonstrated that *RAP2A* and ADRB3 were still strong predictors of BMI (standardized [STD] β = 0.428 and STD β = –0.142, respectively; Supplemental Figure 10, A and B). The variation in BMI explained by the expression of these 5 genes in subcutaneous abdominal adipose was nearly 40% (*R2* = 0.394 for the entire model; Supplemental Figure 10B). Collectively, gene expression from subcutaneous abdominal adipose tissue of 2 independent cohorts (one with men and one with women) of lean and obese human subjects showed strong gene-gene and gene-anthropometric measurement relationships, supporting our model (Figure 6).

## Discussion

Adipocytes play an important role in controlling energy storage during feeding and energy expenditure during fasting, acting by increasing lipolysis and fatty acid oxidation through the actions of epinephrine/norepinephrine on β-ARs. While these anabolic and catabolic signals are normally balanced to ensure energy homeostasis, over- or undernutrition produces broad hormone insensitivity in adipocytes, including resistance to β-adrenergic stimulation (6, 12–14, 34, 56), thus ensuring that adipocytes maintain energy storage even under pathological conditions. Therefore, determining the mechanisms responsible for catecholamine resistance is critically important.

The rate-limiting step in epinephrine/norepinephrine action involves binding to β-ARs at the cell surface. While 3 isotypes of this receptor have been identified, the β3-AR encoded by *Adrb3* is expressed at levels higher by orders of magnitude than those encoded by *Adrb1* or *Adrb2*, making it the predominant receptor subtype in murine adipocytes. In contrast, expression levels of the 3 isotypes are variable in humans (21, 22, 57, 58). Similarly to what was seen in earlier work in C57BL/6 mice (13), we found that adipocytes derived from mice fed HFD were markedly resistant to a synthetic β3 adrenergic agonist. Furthermore, acute administration of the agonist reduced sensitivity of adipocytes to further stimulation both in vivo and in vitro. Additionally, treatment of adipocytes with TNF-α also reduced catecholamine sensitivity, mimicking the impact of HFD. The mechanisms responsible for catecholamine resistance in adipocytes remain incompletely understood. Recent work has implicated ALK7 (56) and the noncanonical NF-κB kinases IKKε and TBK1 (34). However, we demonstrate here that catecholamine resistance, observed in vivo and in vitro, is accompanied by a dramatic reduction in expression of *Adrb3* mRNA and β3-AR protein, suggesting that receptor downregulation is likely to be the key molecular event in catecholamine resistance.

Our studies revealed that β3-AR activation with CL-316243 stimulates the small GTPase RAP2A via activation of EPAC. Once activated, RAP2A stimulates PI-PLC (50). Interestingly, PI-PLC is also activated by norepinephrine via *Adra1*, suggesting that the downregulation of *Adrb3* may be particularly susceptible to endogenous ligand exposure. We show that activation of EPAC/RAP2A stimulates PI-PLC to increase cytosolic calcium (which may be potentiated after norepinephrine exposure), in turn leading to induction of the pseudokinase *Trib1*. *TRIB1* in turn enhances the proteasomal degradation of CEBPα, the key transcriptional activator of *Adrb3* (53). Interestingly, heterologous desensitization produced by inflammatory stimuli activates the same pathway to repress β3-AR expression. Confirming the physiological relevance of this pathway, injection of obese mice with ESI-09, a highly specific inhibitor of EPAC, restored catecholamine signaling in adipocytes and led to weight loss through enhanced energy expenditure. Consistent with these data, global EPAC1 knockout conferred some protection from diet-induced obesity (59). While aP2-Cre–driven EPAC1-knockout mice had the opposite phenotypes (44), this model is not adipocyte specific, and thus it is difficult to interpret these data.

The RAP proteins are members of the Ras subfamily of small GTPases with divergent activation kinetics and roles in cellular functions (49, 60). RAP2A has low GTPase activity, even in the presence of GAPs, and subsequently, a high percentage of the protein is GTP bound in the basal state (49, 60). Thus, overexpression of WT RAP2A was sufficient to decrease CEBPα and downregulate β3-AR in vitro. Because RAP2A and TRIB1 levels are increased with obesity in mice and humans, the basal activity of the G protein may drive *Trib1* induction and subsequent downregulation of β3-AR in vivo. RAP2A can also activate atypical MAPKs, such as TNIK, MINK, and MAP4K4, stimulating JNK and other downstream kinases (61–64), suggesting that RAP2A may have functions beyond desensitization of the β3-AR in healthy adipocytes and in the context of obesity.

We have identified calcium as an important regulator of β3-AR expression via its induction of *Trib1*. Recent work has implicated NFAT5 in the regulation of β3-AR (65). NFATs are generally activated by calcium through the calcineurin phosphatase. While NFAT5 lacks the calcineurin-binding motif (66), NFATc1 was induced by TNF-α treatment in our RNA-Seq data from 3T3L1 adipocytes, and NFATc4 was activated by TNF-α or CL-316243 in vitro (data not shown). Therefore, NFATs may have a role in the regulation of β3-AR expression, possibly as a link between calcium and *Trib1* induction. Although *Trib1* has not been previously investigated in mature adipocytes, GWAS studies have linked *Trib1* to plasma lipid levels (67, 68). This connection was later attributed to changes in liver lipogenesis (52). However, it remains possible that the association in these GWAS studies may result from altered regulation of adipocyte lipolysis by *Trib1’s* role in catecholamine signaling. It is currently unknown how increasing cytosolic calcium leads to *Trib1* induction; however, CEBPB can be induced by calcium (69). Mining of ChIP-Seq data from the Signaling Pathway Project revealed high occupancy of the *Trib1* promoter by CEBPB in all species/all tissues (https://www.signalingpathways.org/ominer/query.jsf?geneSearchType=gene&findMax=y&gene=TRIB1&spesspe=all&reportsBy=pathways&omicsCategory=cistromics&countMax=3000). Furthermore, both TNF-α and CL-316243 in vitro and obesity in vivo induced *Cebpb* in our RNA-Seq data sets, suggesting that CEBPB may be driving *Trib1* induction.

The sensitivity of adipose tissue lipolysis to epinephrine can predict future weight gain and BMI in obese subjects (19, 20). Human abdominal subcutaneous adipose tissue expression of *ADRB3*, *RAP2A*, and *TRIB1* correlated with BMI and inflammatory gene expression to support our in vitro and mouse studies. Consistent with these findings, data (Type Two Diabetes Knowledge Portal; https://t2d.hugeamp.org/) from hundreds of human sequencing studies demonstrates clear correlations between gene polymorphisms in *ADRB3*, *RAP2A*, or *TRIB1* and anthropometric factors, such as weight, obesity, BMI, waste-hip ratio, plasma lipid levels, and type 2 diabetes. These observations, together with our findings that treatment with β3-AR agonists or TNF-α produced RAP2A induction/activation and repression of catecholamine sensitivity through downregulation of the β3-AR, suggest that these pathways are likely to be important in energy metabolism and conserved in obese patients.

Weight-loss therapies have been largely unsuccessful due to low adherence and unwanted side effects (70). Weight-loss strategies may be less effective in obese individuals due to compensations in energy expenditure resulting from caloric restriction or exercise (71, 72). Increasing energy expenditure via β3 agonists has been explored clinically and has shown some positive results in healthy and obese humans (58, 73–76). However, the effects are modest and short lived, limiting their utility (77, 78). We propose that in the obese state, β3-AR expression levels are already reduced by chronic low-grade inflammation, thus limiting the beneficial effects of β3-adrenergic agonists. Furthermore, treatment with a β3-adrenergic agonist results in homologous desensitization, which may explain problems with the durability of these drugs (77, 78). The discovery of this pathway’s function (Figure 6) in adipocytes opens up potential for enhancing the therapeutic effects of agents that increase β-adrenergic activity with fewer unwanted side effects.

## Methods

### Chemicals and reagents.

The following chemicals and reagents were used: 10 μM CL-316243 (MilliporeSigma, C5976) in cells unless otherwise specified and 0.1 mg/kg in mice, 17 ng/mL TNF-α (MilliporeSigma, T7539-50 μg), 1–10 μM ESI-09 (cell culture APExBIO, B4814-5.1), 10 mg/kg ESI-09 (in vivo, MilliporeSigma, 5.00506.0001), Lipofectamine RNAiMAX (Life Technologies, 13778030), 5 μM U73122 (Tocris, 1268), 5 μg/mL cycloheximide (MilliporeSigma, 239765), 50 μM BAPTA-AM (Fisher, 50-136-4911), 4 μM IKK16 (Fisher, IKK INHIBITOR VII/10MG NC1291993), 0.005–50 μM FSK (SellekChem, S2449), Fura2-AM (MilliporeSigma, F0888-5MG), 0.001–10 μM dobutamine (Tocris, 051550), and 0.0001–10 μM formoterol (Tocris, 144810).

### Human subjects.

Human subjects for the above studies were characterized previously (79, 80).

### Animals.

WT mice were C57BL/6J (The Jackson Laboratory) male mice fed a 45% or 60% HFD (Research Diets, D12492) for 16 to 18 weeks starting at 3 months of age. We injected 10 mg/kg ESI-09 1 or vehicle (10% tween 80, 10% ethanol/PBS) i.p. once daily for 7 days after 18 weeks of HFD. Diversity outbred mice derived from 8 different mouse strains have been previously described (54, 55). All mice were put on a 12-hour light/12-hour dark cycle with ad libitum food and water.

### Lipolysis measurements.

FFA levels were measured using either 10 μl of serum or 30 μl of conditioned medium (phenol-free DMEM) with the NEFA Kit (WAKO), using 75 μl reagent/solvent A (999-34691/995-34791) and 150 μl reagent/solvent B (991-34891/993-35191). Absorbance was measured at 550 nm (corrected at 660 nm) using the manufacturer’s protocol. Free glycerol concentration in conditioned media was measured similarly; 20 μl of conditioned pheno-free DMEM was added to 180 μl Free Glycerol Reagent (MilliporeSigma, F6428) and absorbance measured at 540 nm.

### Cell culture.

3T3L1 fibroblasts (ATCC) were cultured in NBCS media (DMEM high glucose [4.5g/L], 10% newborn calf serum [NBCS], 10 U ml penicillin, 10 U ml streptomycin, and 292 mg l glutamine). Fibroblasts were grown to confluency and differentiation commenced 2 days later. Adipocyte differentiation was initiated by addition of 0.5 mM 3-isobutyl-1-methylxanthine (IBMX), 250 nM dexamethasone, and 1 μg ml insulin to FBS media (identical to NBCS media, but with 10% FBS instead of NBCS). After 3 days of this cocktail, adipocytes were switched to FBS media containing 1 μg ml–1 insulin for an additional 3 days. Then media was switched to FBS media, and cells were assayed 2 to 4 days later. Only cultures with more than 90% of cells displaying adipocyte morphology were used in all assays. The SVF isolation procedure has been previously described (81). In brief, SVF was isolated from iWAT of WT C57BL/6J (Jackson Laboratory) mice and was cultured similarly with slight modifications. SVF was cultured in DMEM/F12 with 15% FBS and 2 μg/mL amphotericin (MilliporeSigma, A2411). Dexamethasone concentration was 5 μM, and 1 μM troglitazone was added during differentiation. Resulting differentiated adipocytes are referred to as PPDIVs.

### Cell-based mitochondrial respiration.

Oxygen-consumption rates were measured using a Seahorse XF96 analyzer. SVF was differentiated on a 96-well Seahorse plate. Pretreatments with 0.1 μM CL-316243 or 17 ng/mL TNF-α were performed 18 hours before the start of the assay. On the test day, cells were washed with base media (DMEM containing 8 mM glucose, 1 mM pyruvate, 2 mM glutamine, and 0.5 mM carnitine without phenol red or sodium bicarbonate), then incubated in a CO_2_-free incubator for 15 minutes prior to measurement of oxygen consumption. CL-316243 was injected at a final concentration of 1 μM, oligomycin at 2 μM, FCCP at 1 μM, and rotenone and antimycin A at 1 μM each.

### Metabolic cage studies.

Mice fed HFD for 16 weeks were injected once daily for 1 week with vehicle while simultaneously acclimating to the metabolic chambers (Promethium Systems) and were housed in a temperature-controlled cabinet. The metabolic chambers measured VO_2_, VCO_2_, activity (meters moved), and cumulative food consumption during the second week in the metabolic cages when half the mice were switched to once-daily injections of ESI-09 at 10 mg/kg body weight.

### Histology.

iWAT and eWAT were collected and fixed in 10 % buffered formalin for 72 hours, then washed and stored in 70% ethanol until processing. Paraffin embedding, sectioning, H&E staining, and IHC for UCP1 (antibody from Abcam, catalog ab10983) were conducted at the UCSD Tissue Technology Core in the Moores Cancer Center.

### Live-cell imaging.

Detection of calcium mobilization by Fura2-AM was conducted as previously described (82). Briefly, 3T3L1 adipocytes were serum starved and pretreated with 10 μM ESI-09. Cells were then loaded with Fura2-AM before stimulation with 10 μM CL316,243 or 17 ng/mL TNF-α for 30 minutes. Time courses were averaged from normalized Fura2 ratios (F340/380). Cells were randomly chosen, and experiments were repeated 3 to 4 times.

### Rap activity assay.

The rap activity assay was performed using Rap1 Assay Reagent (Ral GDS-RBD, agarose), 650 μg, from MilliporeSigma (14-455), according to the manufacturer’s protocol, with slight modification. Lysis and wash buffer contained 50 mM Tris pH 7.5, 150 mM NaCl, 2 mM EDTA, 10% glycerol, 1% NP-40, and 10 mM magnesium chloride. Protease III inhibitor cocktail (Millipore, 539134), phosphatase II inhibitor cocktail (MilliporeSigma, P5726) and phosphatase III inhibitor cocktail (MilliporeSigma, P0044) were added to the lysis buffer at 1:100 dilution just prior to lysis. For Western blotting Rap1 antibody (CST, catalog 4938s) and Rap2 antibody (BD, catalog 610215) were used. Note that the interaction between RAP and Ral GDS-RBD is lost rapidly after lysis and that incubation with beads should occur within 1 hour of lysis.

### Western blotting.

Tissues were homogenized with a tissue grinder and cells with a 31-gauge needle. RIPA buffer was used for cell lysis and contained 50 mM Tris pH 7.5, 150 mM NaCl, 0.1% SDS, 0.5% deoxycholate, and 1% NP-40. Protease III inhibitor cocktail (Millipore, 539134), phosphatase II inhibitor cocktail (MilliporeSigma, P5726,5 mL) and phosphatase III inhibitor cocktail (MilliporeSigma, P0044, 5 mL) were added to the lysis buffer at 1:100 dilution just prior to lysis. Homogenates were centrifuged at 17,000*g* for 25 minutes, then transferred to a fresh tube, and protein concentration was measured using the BCA assay method. Equal protein was denatured in sample buffer and resolved with SDS–PAGE. Samples were transferred to nitrocellulose membranes with a pore size of 0.22 μm (Bio-Rad). Individual proteins were detected using 1:1000 dilution of primary antibodies, as follows: Rap1 (Cell Signaling Technology [CST], catalog 4938), Rap2 (BD, catalog 610215), β3-AR (Abcam, catalog ab94506) (note samples were not boiled for Western blot when blotting for β3-AR), HSP90 (CST, catalog 4877), HSL (CST, catalog 4107), pHSL_563_ (CST, catalog 4137), pHSL_660_ (CST, catalog 4126s), CEBPα (CST, catalog 8178), EPAC1 (CST, catalog 4155), FLAG rabbit (CST, catalog 14793) UCP1 (Abcam, catalog ab10983) pP38 (CST, catalog 9211s), p38 (CST, catalog 9212s), and α-tubulin (CST, catalog 3873). Proteins were visualized on film with horseradish peroxidase–conjugated secondary antibodies diluted 1:10,000 (Pierce).

### cAMP assay.

The Direct cAMP Enzyme Immunoassay Kit (CA200-1KT) was purchased from MilliporeSigma, and cAMP levels were measured according to the manufacturer’s protocol.

### qPCR.

RNA extractions from iWAT and eWAT were performed using TRIzol reagent (Life Technologies, 15596018) followed by purification using the PureLink RNA Mini Kit (Ambion, 12183025). Reverse transcription was carried out using the Applied Biosystems High-Capacity cDNA Reverse Transcription Kit (catalog 43-688-13). Quantitative PCR amplification was performed with Power SYBR Green PCR Master Mix (Applied Biosystems, catalog 43-676-59) using the Applied Biosystems QuantStudio 5 Real-Time PCR System. All samples were run in triplicate. Primer sequences were as follows: *Arbp* (forward CACTGGTCTAGGACCCGAGAA; reverse CACTGGTCTAGGACCCGAGAA); *Adrb3* (forward, GGCCCTCTCTAGTTCCCAG; reverse TAGCCATCAAACCTGTTGAGC); *Trib1* (forward, AGAACCCAGCTTAGACTGGAA; reverse AAAAGCGTATAGAGCATCACCC); *Tnf* (forward, CCCTCACACTCAGATCATCTTCT; reverse GCTACGACGTGGGCTACAG); *Rap2a* (forward, AATACGACCCCACCATCGAG; reverse ACCTTCTCATACCGCTTCACG); *Adrb1* (forward, CTCATCGTGGTGGGTAACGTG; reverse ACACACAGCACATCTACCGAA); *Adrb2* (forward, GGGAACGACAGCGACTTCTT; reverse GCCAGGACGATAACCGACAT); *Ucp1* (forward, AGGCTTCCAGTACCATTAGGT; reverse CTGAGTGAGGCAAAGCTGATTT); *Dio2* (forward, AATTATGCCTCGGAGAAGACCG; reverse GGCAGTTGCCTAGTGAAAGGT); *Cidea* (forward, TGACATTCATGGGATTGCAGAC; reverse GGCCAGTTGTGATGACTAAGAC); and *Prdm16* (forward, CCCCACATTCCGCTGTGAT; reverse CTCGCAATCCTTGCACTCA).

### RNA-Seq analysis.

RNA extraction from 3T3L1 adipocytes was conducted similarly to the qPCR protocol above. One sample from 3 independent experiments was used for each condition (*n* = 3/group). RNA (100–500 ng) was utilized for library preparation with the TruSeq Stranded mRNA Kit (Illumina) according to the manufacturer’s protocol. Libraries were validated using a 2100 BioAnalyzer (Agilent), then normalized and pooled for sequencing using bar-coded multiplexing at a 90 bp single-end read length on an Illumina HiSeq 4000. Samples were sequenced to a median depth of 14 million reads, and fastq files were generated automatically using Illumina bcl2fastq2 Conversion Software. Read alignment and junction mapping to genome build GRCh38 was accomplished using STAR, version 2.7.2b (83). Known splice junctions from mm10 were supplied to the aligner, and de novo junction discovery was also permitted. Differential gene expression analysis and statistical testing were performed using Cuffdiff2, version 2.2.1 (84), employing the Ensembl genome annotation. Transcript expression was calculated as gene-level relative abundance in fragments per kilobase of exon model per million mapped fragments. Venn diagrams were generated using Venny (https://bioinfogp.cnb.csic.es/tools/venny/).

### Plasmids and viral constructs.

pcDNA3 plasmids containing Flag-Cebpa (66978), Flag-Trib1 (131156), Flag-Rap1b N17 (118322), and Flag-Rap1b V12 (118323) were purchased from Addgene. Plasmids containing Rap2a (55666) and Rap2b (55667) were also purchased from Addgene. Plasmids containing Rap2a and Rap2b genes were transferred to the pcDNA3 vector and mutated to dominant=negative N17 and constitutively active V12 using the In-fusion HD EcoDRy Cloning Kit from Takara (639690) according to the manufacturer’s protocol. Furthermore, *Rap2a* mutants, *Cebpa*, and *Trib1* were cloned into pLVX for lentiviral production. Lentivirus particles were produced in HEK293T LentiX cells (ATCC) as previously described (81) and used to treat 3T3L1 and PPDIVs.

### Electroporation experiments.

Electroporation was conducted in 10 cm plates containing 3T3L1 adipocytes. Adipocytes were electroporated on differentiation day 6 or 7, and each 10 cm plate was seeded into one-half of a 12-well plate. A Bio-Rad electroporation machine and cuvettes were used. Each electroporation consisted of cells from a 10 cm plate and 200 μg plasmid of plasmid DNA. The electroporation machine was set to volts (kV) to 0.160 and high CAP (μF × 1000) to 0.950, with the capacitance (μF) knob pointed at high CAP (500 V max). Cells were allowed 2 days for expression of vectors before commencement of experiments.

### siRNA experiments.

siRNA experiments were conducted using Lipofectamine RNAiMAX according to the manufacturer’s protocol, with small modifications. Adipocytes on day 5 of differentiation were treated with 60 pmole siRNA and 3 μL of lipofectamine for 1 well of a 24-well plate. siRNAs were SMARTpool ON-TARGETplus from Dharmacon (Trib1, L-057134-00-0005; Rap2a, L-041528-01-0005; and Nontargeting pool, D-001810-10-05). Media was not changed after transfection, and experiments were conducted 48 hours after transfection.

### Statistics.

All statistical analyses were 2 sided, with normality, heterogeneity, and independent samples assumed unless otherwise stated. Two-group comparisons were conducted with Student’s *t* test. For analysis of 2 groups, ANOVA was utilized, and when more than 1 independent variable was analyzed, higher order ANOVAs were used with Tukey’s honest significance test (HSD) or least significant difference (LSD) test, or Dunnett’s or Šidák’s post hoc comparisons. Simple or multipule linear regression analyses were performed for gene-gene and gene-anthropometric measurement and correlations. Statistical analyses were performed using SPSS, version 26, and GraphPad Prism, version 9.

### Study approval.

All animal experiments with the exception of those using DO mice were approved by the IACUC of UCSD. Animal care and study protocols for DO mice were approved by the University of Wisconsin–Madison Animal Care and Use Committee as previously described (54–54). Approval for all human studies has been described previously (79–80). In brief, studies using female subjects were approved by the ethical committee at the Karolinska University Hospital. All subjects were informed in detail about the studies, and written informed consent was obtained. Studies conducted in male subjects were approved by the ethics committee of the Northern Savo Hospital District, and all participants gave written informed consent.

## Author contributions

JMV designed and conducted experiments, analyzed and interpreted data, prepared figures, and wrote the manuscript. MA, OK, MAO, PZ, MPK, AJL, RTY, CL, MD, ADA, JZ, XZ, HG, RME, and MR designed and conducted experiments, analyzed and interpreted data, and edited the manuscript. ARS directed the project, designed experiments, analyzed and interpreted data, prepared figures, and edited the manuscript.

## Supplementary Material

Supplemental data

## Version 1 11/30/2021

In-Press Preview

## Version 2 01/18/2022

Electronic publication
