# Supplementary material for: β3-Adrenergic receptor downregulation leads to adipocyte catecholamine resistance in obesity
Source: J Clin Invest. 2022 Jan 18;132(2):e153357. doi: 10.1172/JCI153357 (PMC8759781; doi:10.1172/JCI153357)
Supplement: Supplemental data [file jci-132-153357-s193.pdf]

Figure s1

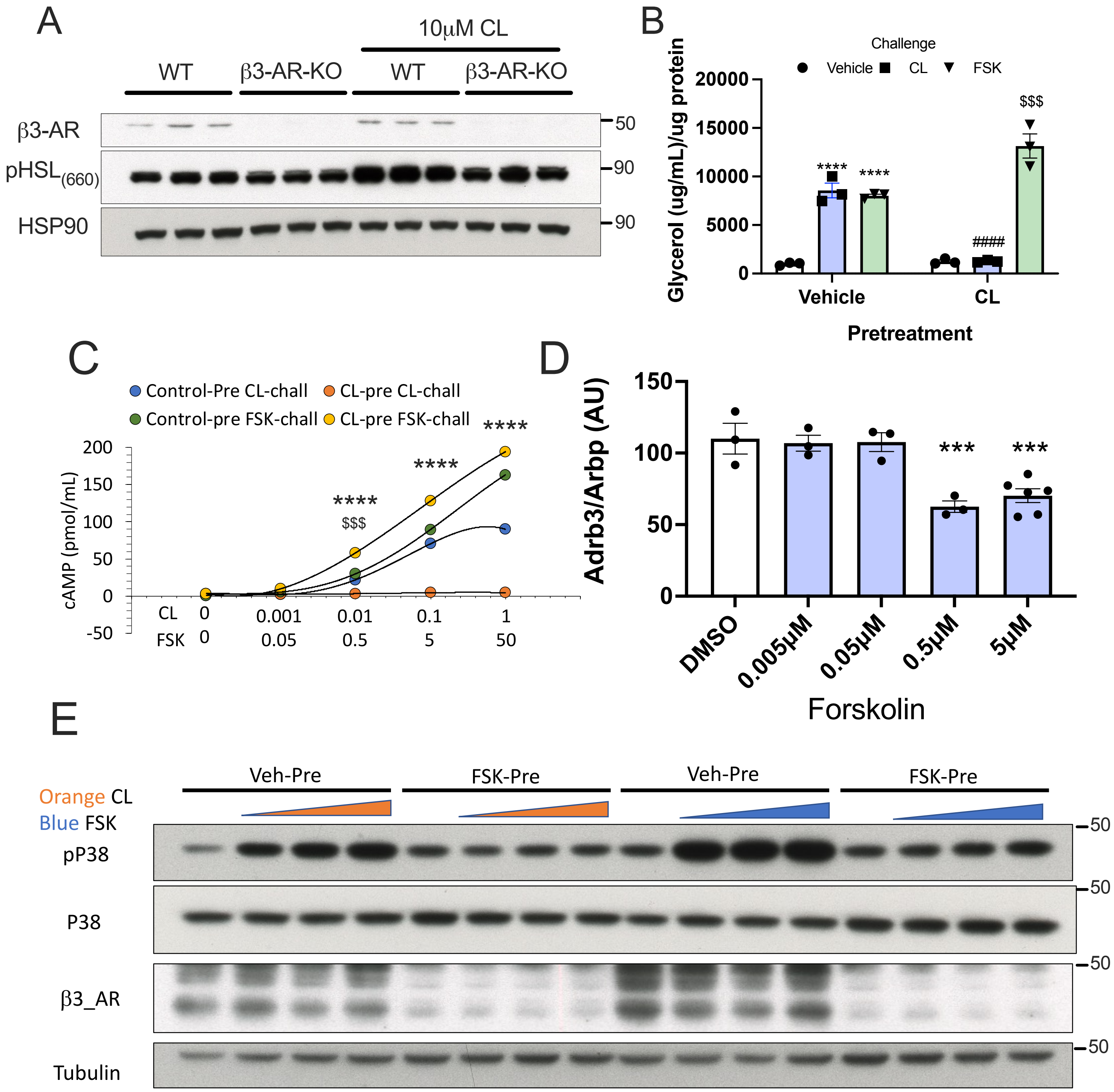
A
10μM CL
WT
β3-AR-KO
WT
β3-AR-KO
β3-AR
pHSL(660)
HSP90
50
90
90
B
Challenge
Vehicle
CL
FSK
Glycerol (ug/mL)/ug protein
20000
15000
10000
5000
0
****
****
$$$
####
Vehicle
CL
Pretreatment
C
Control-Pre CL-chall
CL-pre CL-chall
Control-pre FSK-chall
CL-pre FSK-chall
cAMP (pmol/mL)
200
150
100
50
0
-50
****
$$$
****
****
CL 0 0.001 0.01 0.1 1
FSK 0 0.05 0.5 5 50
D
Adrb3/Arbp (AU)
150
100
50
0
***
***
DMSO
0.005μM
0.05μM
0.5μM
5μM
Forskolin
E
Orange CL
Blue FSK
Veh-Pre
FSK-Pre
Veh-Pre
FSK-Pre
pP38
P38
β3_AR
Tubulin
50
50
50
50

Figure S1: (**A**) β3-AR protein expression and pHSL assessed in β3-AR knockout adipocytes. CL-316243 treatment was 15mins. Supplemental Figure 1A utilized 3T3L1 adipocytes that were CRISPR gene edited to knockout β3-AR. After selection of CRISPR modified preadipocytes, the β3-KO and parent cell lines had reduced differentiation capacity. Despite this, they had sufficient differentiation for β3-AR detection as shown as a single band by western blotting after 1-hour exposure with 20ug of protein loaded per lane. In contrast, 3T3L1 adipocytes used in all other experiments (from ATCC) in the manuscript were capable of full differentiation and subsequently had orders of magnitude higher expression of β3-AR (identical conditions and immediate exposure) as shown by the multiple banding pattern. β3-AR is post translationally modified by phosphorylation and palmitoylation (PMID: 29661693 and PMID: 30541923), resulting in different pools of β3-AR that run at different speeds on the gel causing the multiple banding pattern. We suspect that this multiple banding pattern is not visible in the β3-KO cells because β3-AR was not expressed at high enough levels to detect these other bands. (**B**) 3T3L1 adipocytes were pretreated for 48hrs with 10μM CL-316243 and challenged with 1μM CL-316243 or 50μM FSK and 60mins later glycerol release determined by absorbance (n=3 per group). * equals significance from vehicle-pre-vehicle-challenge, # equals significance from vehicle-pre- CL-316243-challenge to CL-316243-pre- CL-316243-challenge, and $ equals significance from vehicle-pre-FSK-challenge to CL-316243-pre-FSK-challenge. (**C**) 3T3L1 adipocytes were pretreated for 48hrs with 10μM CL-316243 then challenged with CL-316243 (1μM-0.001μM) or forskolin (FSK) (50μM-0.05μM) and cAMP was measured by immunoassay (n=2 per group). * equals significance from vehicle-pre-vehicle-challenge in all groups except CL-316243-pre- CL-316243-challenge group where no significant differences were observed. $ equals significance of vehicle-pre-vehicle-challenge to vehicle-pre- CL-316243-challenge at that given dose. (**D**) Forskolin dose response curve assessing downregulation of *Adrb3* by qPCR in 3T3L1 adipocytes (n=3-6 per group). (**E**) 3T3L1 adipocytes were pretreated for 48hrs with 5μM forskolin and challenged with increasing concentration of CL-316243 (0.01μM-1μM) or FSK (0.5μM-50μM), then β3-AR and phosphorylation of P38 determined by western blot (representative of n=3 per group). (**B-C**) was calculated with 2-way ANOVAs with Tukey and Sidak post hocs respectively. (**C**) was calculated with a one-way ANOVA with Dunnett's posthoc. * equals significance from control unless otherwise specified. All error bars are standard error of the mean.

Figure s2

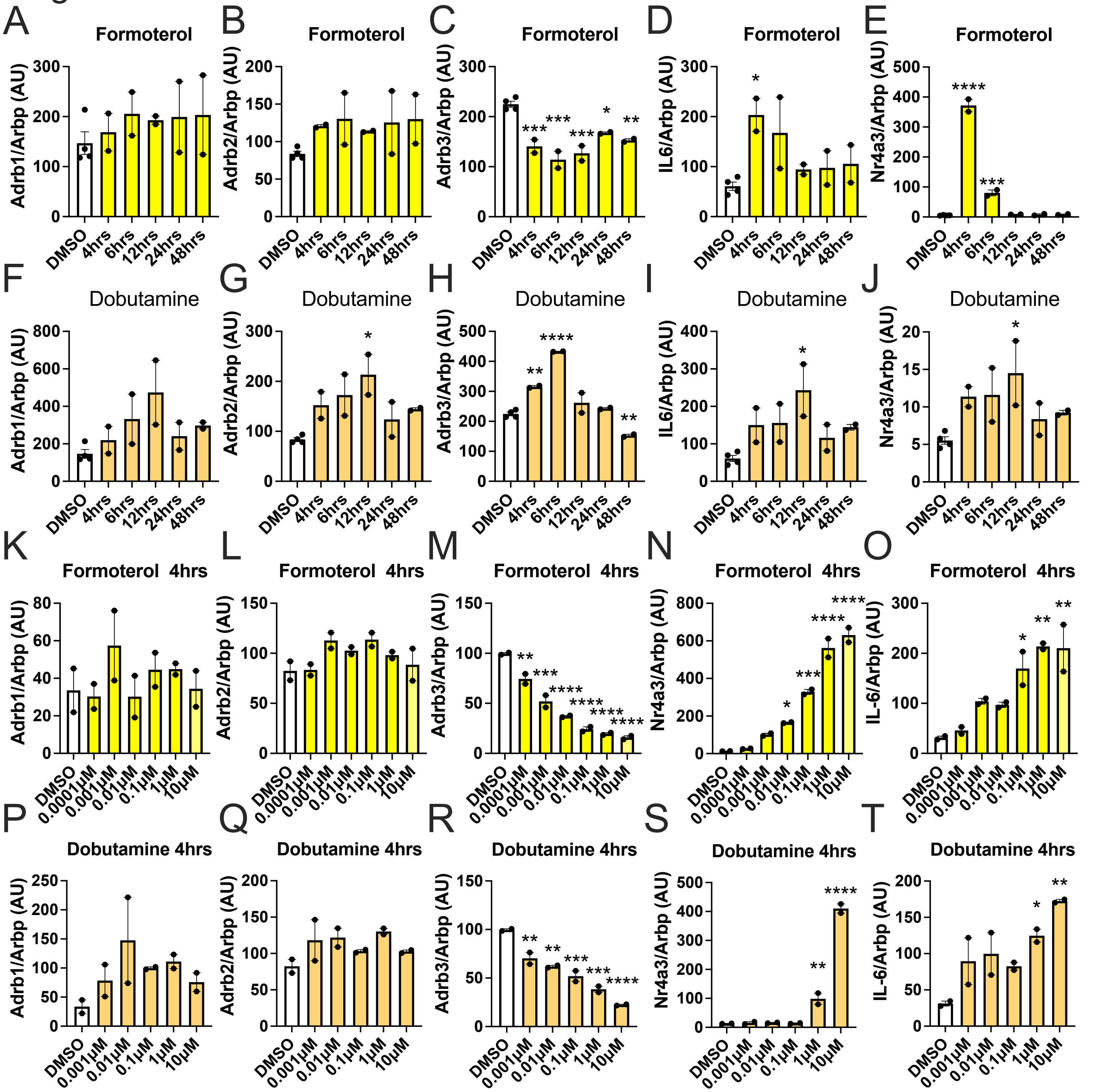
A
Formoterol
Adrb1/Arbp (AU)
B
Formoterol
Adrb2/Arbp (AU)
C
Formoterol
Adrb3/Arbp (AU)
D
Formoterol
IL6/Arbp (AU)
E
Formoterol
Nr4a3/Arbp (AU)
DMSO
4hrs
6hrs
12hrs
24hrs
48hrs
F
Dobutamine
Adrb1/Arbp (AU)
G
Dobutamine
Adrb2/Arbp (AU)
H
Dobutamine
Adrb3/Arbp (AU)
I
Dobutamine
IL6/Arbp (AU)
J
Dobutamine
Nr4a3/Arbp (AU)
K
Formoterol 4hrs
Adrb1/Arbp (AU)
L
Formoterol 4hrs
Adrb2/Arbp (AU)
M
Formoterol 4hrs
Adrb3/Arbp (AU)
N
Formoterol 4hrs
Nr4a3/Arbp (AU)
O
Formoterol 4hrs
IL-6/Arbp (AU)
0.0001µM
0.001µM
0.01µM
0.1µM
1µM
10µM
P
Dobutamine 4hrs
Adrb1/Arbp (AU)
Q
Dobutamine 4hrs
Adrb2/Arbp (AU)
R
Dobutamine 4hrs
Adrb3/Arbp (AU)
S
Dobutamine 4hrs
Nr4a3/Arbp (AU)
T
Dobutamine 4hrs
IL-6/Arbp (AU)

Figure S2: (**A-J**) Time course up to 48hrs in 3T3L1 adipocytes treated with β2-AR specific agonist formoterol (0.1μM) or β1-AR agonist dobutamine (1μM). β-AR gene (Adrb1, adrb2 and adrb3) and cAMP regulated gene (Il6 and Nr4a3) expression were determined by qPCR (n=2 per group). (**K-T**) dose response curves in 3T3L1 adipocytes treated with β2-AR specific agonist formoterol or β1-AR agonist dobutamine for 4hrs. β-AR gene (Adrb1, adrb2 and adrb3) and cAMP regulated gene (Il6 and Nr4a3) expression were determined by qPCR (n=2 per group). (**A-T**) were calculated with one-way ANOVAs and Dunnett's posthoc test. * equals significance from control unless otherwise specified. All error bars are standard error of the mean.

Figure s3

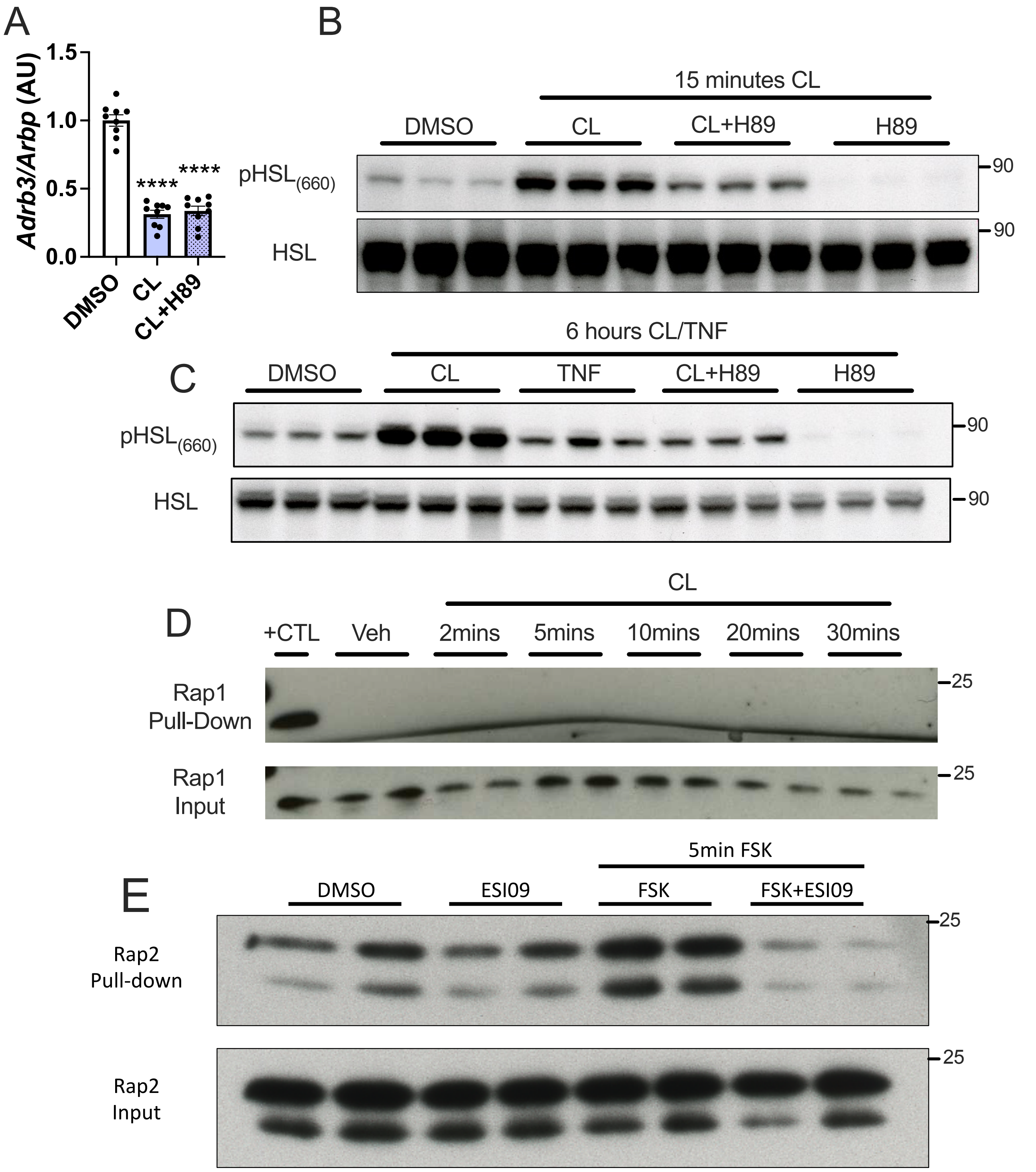
A
Adrb3/Arbp (AU)
1.5
1.0
0.5
0.0
****
****
DMSO
CL
CL+H89
B
15 minutes CL
DMSO
CL
CL+H89
H89
pHSL(660)
HSL
90
90
6 hours CL/TNF
C
DMSO
CL
TNF
CL+H89
H89
pHSL(660)
HSL
90
90
CL
D
+CTL
Veh
2mins
5mins
10mins
20mins
30mins
Rap1 Pull-Down
Rap1 Input
25
25
5min FSK
E
DMSO
ESI09
FSK
FSK+ESI09
Rap2 Pull-down
Rap2 Input
25
25

Figure S3: (**A**) 3T3L1 adipocytes were pretreated with DMSO or PKA inhibitor H89 (20μM) for 1hr then challenged with CL-316243 10μM (n=3 per group from 3 independent experiments) for 3hrs and gene expression measured using qPCR. (**B-C**) 3T3L1 adipocytes were pretreated with DMSO or PKA inhibitor H89 for 1hr then challenged with CL-316243 10μM and pHSL measured by western blotting (n=3 per group). (**D**) Active (GTP bound) RAP1 was determined by pulldown with RalGDS(RBD) agarose beads followed by western blotting with RAP1 specific antibody (n=2 per group). (**E**) Active (GTP bound) RAP2 in response to forskolin 50μM ±ESI-09 10μM was determined by pulldown with RalGDS(RBD) agarose beads followed by western blotting with RAP2 specific antibody (n=2 per group). (**A**) Was calculated using one-way ANOVA with Tukey post hoc comparisons. * equals significance from control unless otherwise specified. All error bars are standard error of the mean.

Figure s4

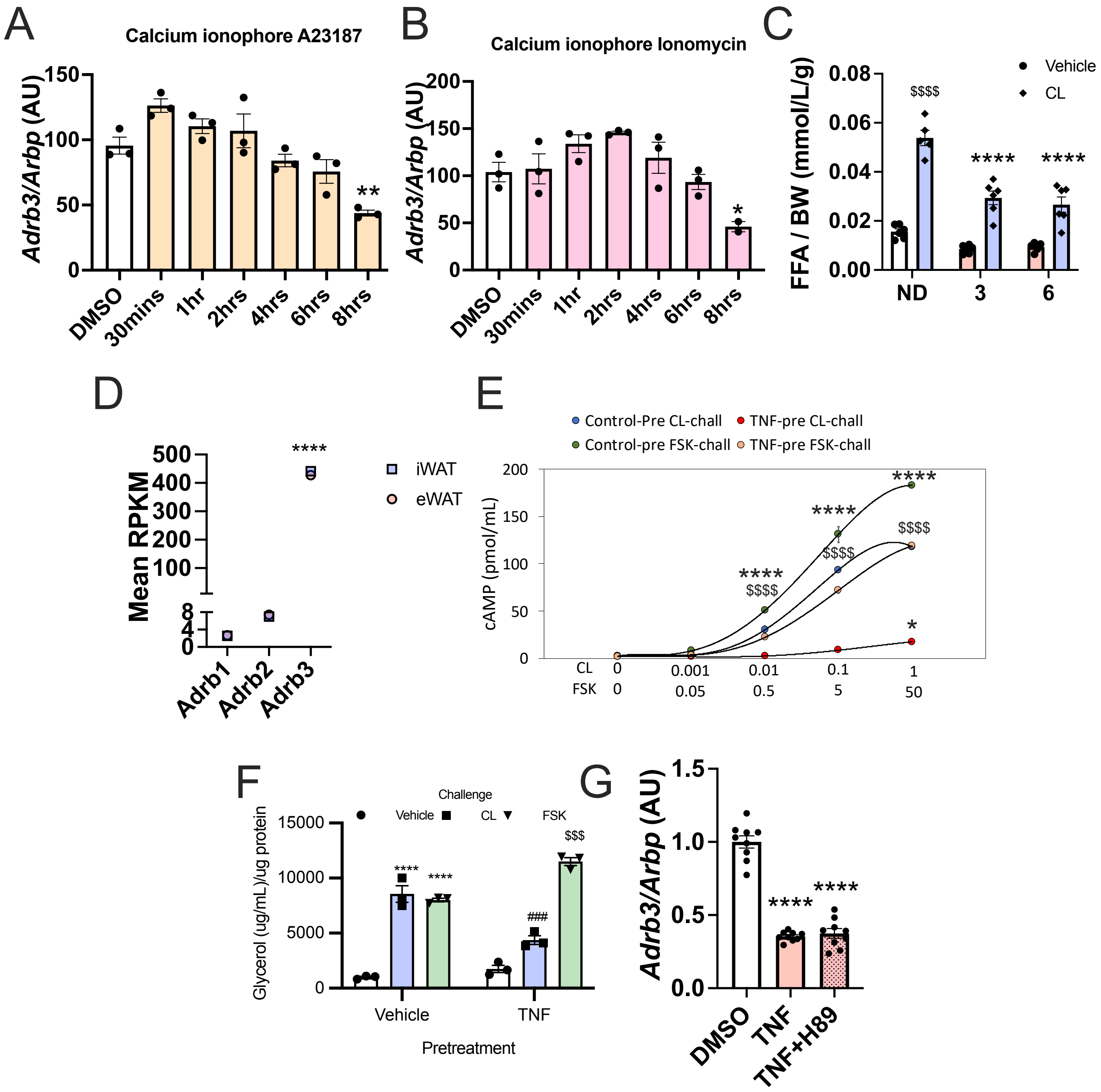
A
Calcium ionophore A23187
Adrb3/Arbp (AU)
DMSO
30mins
1hr
2hrs
4hrs
6hrs
8hrs
B
Calcium ionophore Ionomycin
C
FFA / BW (mmol/L/g)
Vehicle
CL
ND
3
6
D
Mean RPKM
iWAT
eWAT
Adrb1
Adrb2
Adrb3
E
Control-Pre CL-chall
TNF-pre CL-chall
Control-pre FSK-chall
TNF-pre FSK-chall
cAMP (pmol/mL)
CL
FSK
F
Challenge
Vehicle
CL
FSK
Glycerol (ug/mL)/ug protein
Vehicle
TNF
Pretreatment
G
DMSO
TNF
TNF+H89

Figure S4: (**A-B**) 3T3L1 adipocytes were treated with 2μM A23187 or Ionomycin for the indicated time (n=2-3 per group) and qPCR was preformed to assess *Adrb3* expression. (**C**) Mice were placed on HFD for 3 or 12 months (n=3-6 per group), then challenged with 0.5mg/kg CL-316243 and serum FFA normalized to body weight measured. $ equals significance from vehicle and * equals significance compared to ND-CL-316243 (**D**) RNAseq on isolated adipocytes from iWAT and eWAT of mice on a HFD for 16 weeks (n=3 per group). (**E**) 3T3L1 adipocytes were pretreated for 48hrs with 17ng/mL TNFα and challenged as in supplemental figure 1 then cAMP measured by immunoassay (n=2 per group). * equals significance from vehicle-pre-vehicle-challenge in all groups except CL-316243-pre- CL-316243-challenge group where no significant differences were observed. $ equals significance of vehicle-pre-vehicle-challenge to vehicle-pre- CL-316243-challenge at that given dose. (**F**) 3T3L1 adipocytes were pretreated for 48hrs with 17ng/mL TNFα and challenged with 1μM CL-316243 or 50μM FSK and glycerol release was determined as before (n=3 per group). * equals significance from vehicle-pre-vehicle-challenge, # equals significance from vehicle-pre- CL-316243-challenge to CL-316243-pre-CL-316243-challenge, and $ equals significance from vehicle-pre-FSK-challenge to CL-316243-pre-FSK-challenge. Control samples for F are also used in supplemental figure 1B as these experiments were conducted at the same time. (**G**) 3T3L1 adipocytes were pretreated with DMSO or PKA inhibitor H89 (20μM) for 1hr then challenged with 17ng/mL TNFα and *Adrb3* measured by qPCR (n=3 per group from 3 independent experiments). Control samples are also used in supplemental figure 3A as experiments were conducted at the same time. (**A-B**) were calculated using one-way ANOVA with Dunnett's post hoc comparisons. (**C**) Was analyzed by 2-way ANOVA with Sidak multiple comparison. (**D**) Was calculated with t-tests and false discovery rates applied to correct for multiple comparisons. (**E-F**) Were calculated with a 2-way ANOVA and Tukey posthoc. (**G**) Was calculated using one-way ANOVA with Tukey post hoc comparisons. * equals significance from control unless otherwise specified. All error bars are standard error of the means.

Figure s5

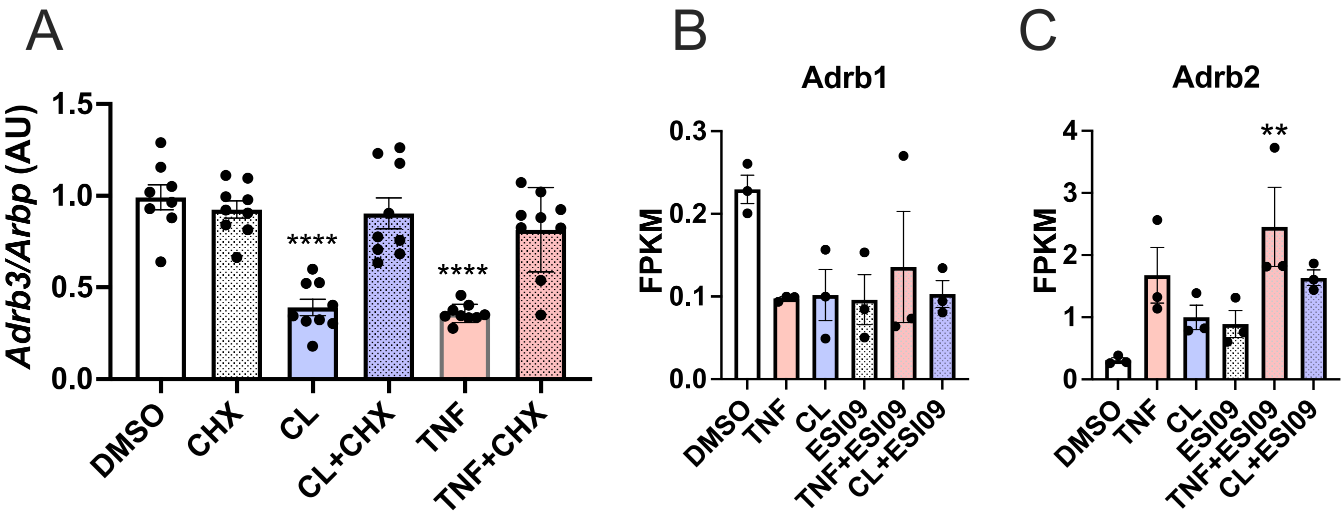

Figure S5: (**A**) 3T3L1 adipocytes were pretreated for 10mins with 10ug/mL cycloheximide (CHX protein synthesis inhibitor) followed by 3hrs challenge with 17ng/mL TNFα or 10μM CL-316243 (n=3 per group from 3 independent experiments) and qPCR was preformed to assess *Adrb3* expression. (**B-C**) RNAseq from 3T3L1 adipocytes treated with 17ng/mL TNFα or 10μM CL-316243 for 3hrs ±ESI-09 10μM (n=3 per group). (**A**) was calculated using one-way ANOVA with Tukey post hoc comparisons. (**B-C**) Were calculated using one-way ANOVA with Dunnett's post hoc comparisons. * equals significance from control unless otherwise specified. All error bars are standard error of the means.

Figure s6

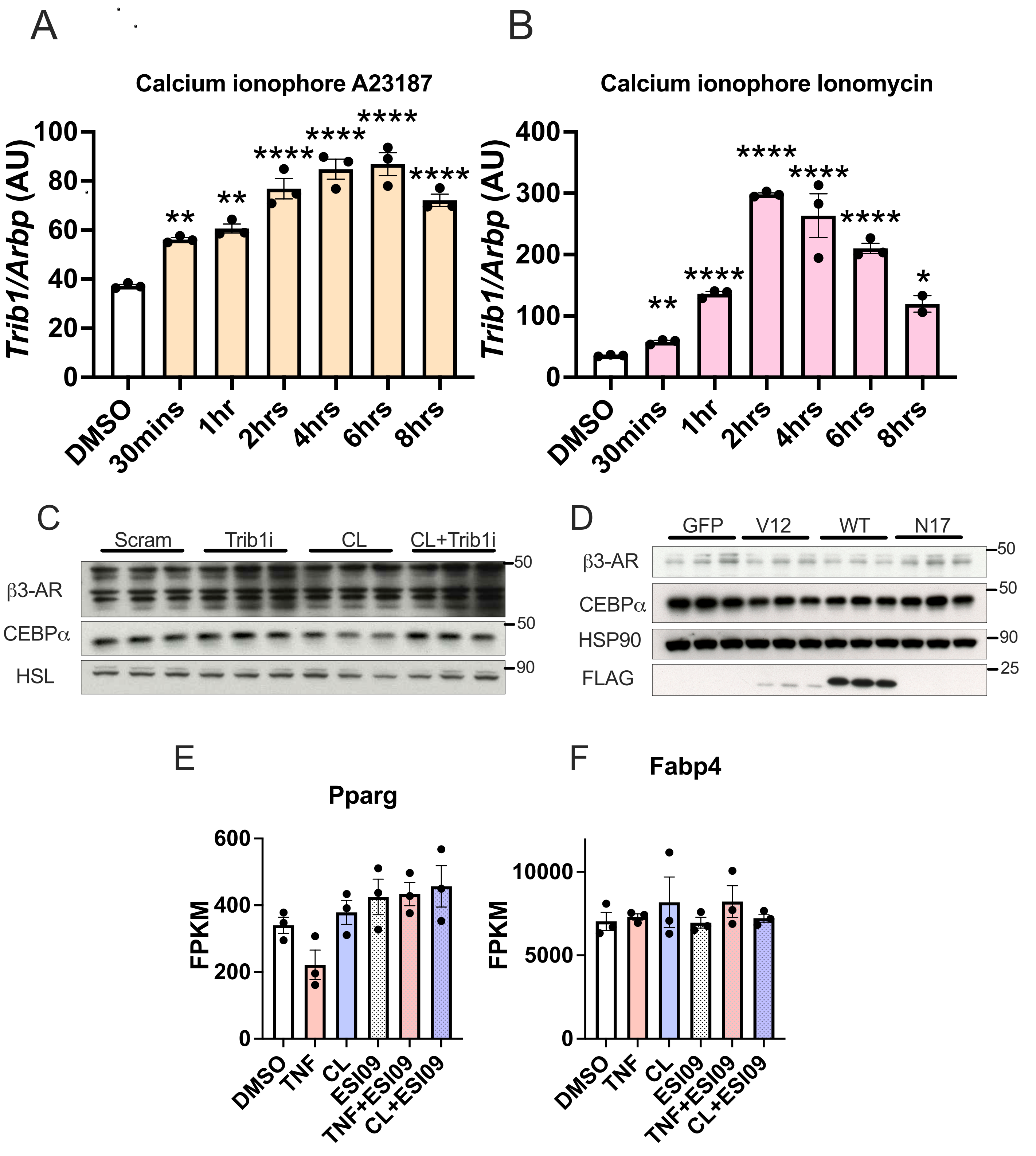
A
Calcium ionophore A23187
Trib1/Arbp (AU)
0
20
40
60
80
100
DMSO
30mins
1hr
2hrs
4hrs
6hrs
8hrs
**
**
****
****
****
****
B
Calcium ionophore Ionomycin
Trib1/Arbp (AU)
0
100
200
300
400
DMSO
30mins
1hr
2hrs
4hrs
6hrs
8hrs
**
****
****
****
****
*
C
Scram
Trib1i
CL
CL+Trib1i
β3-AR
CEBPα
HSL
50
50
90
D
GFP
V12
WT
N17
β3-AR
CEBPα
HSP90
FLAG
50
50
90
25
E
Pparg
FPKM
0
200
400
600
DMSO
TNF
CL
ESI09
TNF+ESI09
CL+ESI09
F
Fabp4
FPKM
0
5000
10000
DMSO
TNF
CL
ESI09
TNF+ESI09
CL+ESI09

Figure S6: (**A-B**) 3T3L1 adipocytes were treated with 2μM A23187 or Ionomycin for the indicated time (n=2-3 per group) and qPCR was preformed to assess *Trib1* expression. (**C**) siRNA against *Trib1* was delivered to 3T3L1 adipocytes and 48hrs later cells were challenged with 1μM CL-316243 or 17ng/mL TNFα for 18hrs (n=3 per group) and β3-AR/CEBPα protein expression assessed by western blotting. (**D**) Primary pre-adipocytes differentiated *in vitro* were transduced with lentivirus containing RAP2A WT, V12 (constitutively active), N17 (dominant negative) or GFP (control) (n=3 per group) and β3-AR/CEBPα protein expression assessed by western blotting 4days later. (**E-F**) RNAseq from 3T3L1 adipocytes treated with 17ng/mL TNFα or 10μM CL-316243 for 3hrs ±ESI-09 10μM (n=3 per group). * equals significance from control unless otherwise specified. (**A-B and E-F**) Were calculated by one-way ANOVA with Dunnett's posthocs. All error bars are standard error of the means.

Figure s7

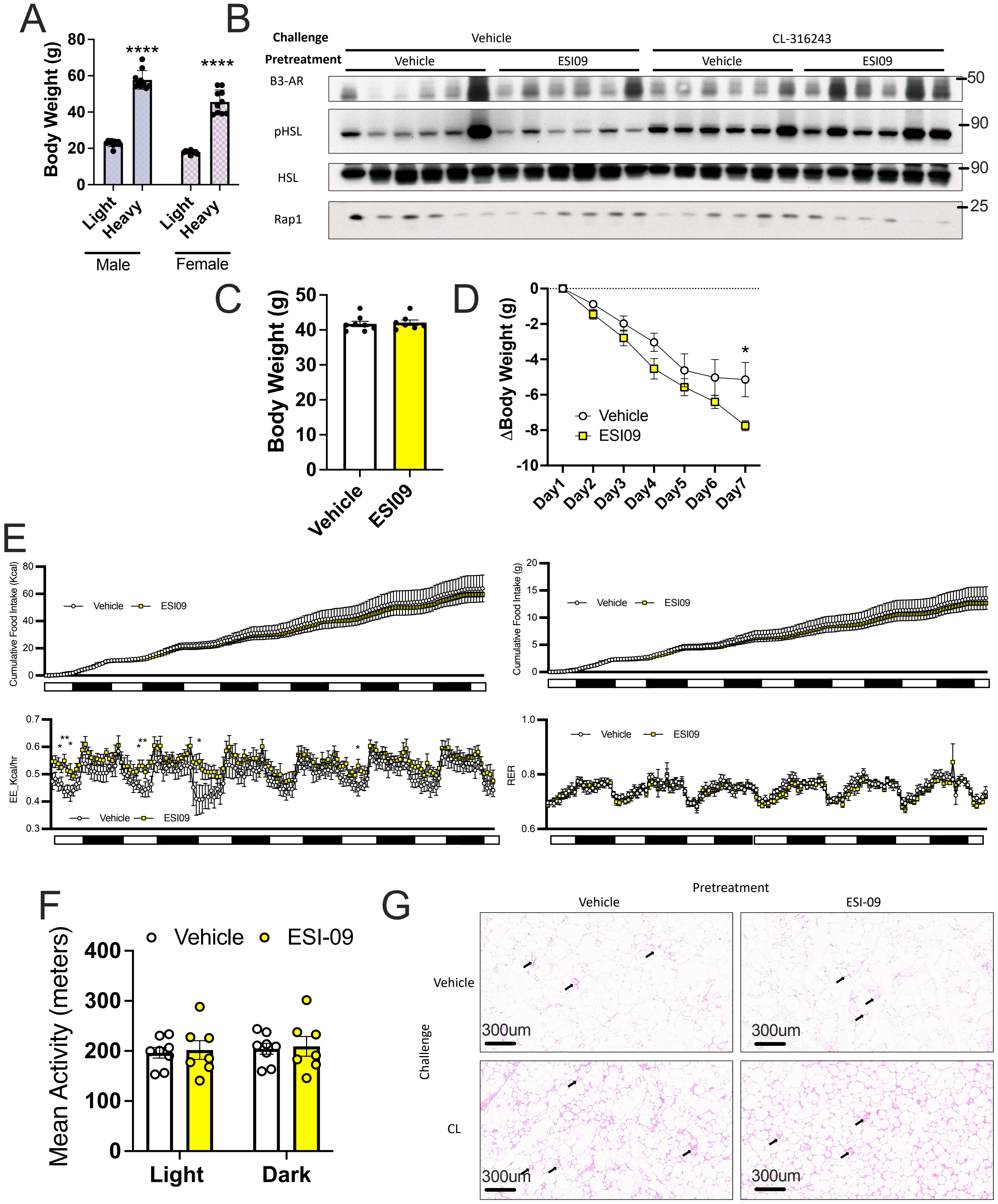
A
Body Weight (g)
80
60
40
20
0
****
****
Light
Heavy
Light
Heavy
Male
Female
B
Challenge
Vehicle
CL-316243
Pretreatment
Vehicle
ESI09
Vehicle
ESI09
B3-AR
pHSL
HSL
Rap1
50
90
90
25
C
Body Weight (g)
50
40
30
20
10
0
Vehicle
ESI09
D
ΔBody Weight (g)
0
-2
-4
-6
-8
-10
*
Vehicle
ESI09
Day1
Day2
Day3
Day4
Day5
Day6
Day7
E
Cumulative Food Intake (Kcal)
Vehicle
ESI09
Cumulative Food Intake (g)
EE_Kcal/hr
RER
F
Mean Activity (meters)
Vehicle
ESI-09
400
300
200
100
0
Light
Dark
G
Pretreatment
Vehicle
ESI-09
Challenge
Vehicle
CL
300um

Figure S7: (**A**) Body Weight at sacrifice of DO mice corresponding to Figure 7A. (**B**) Western blot of β3-AR and pHSL from iWAT of mice (corresponding to Figure 7B-D); 24hrs after ESI09 treatment cessation mice were injected with CL-316243 0.1mg/kg or saline for 20mins and tissues harvested (n=6 per group). (**C**) Initial body weights (**D**) Percent change in body weight for mice in metabolic cage study. (**E**) Whole body EE, cumulative food consumption in grams and kcal, respiratory exchange ratio (RER) and (**F**) mean activity expressed as total meters moved in light and dark phases in a second cohort of 16 week HFD fed mice treated with or without ESI-09 for 1 week (n=7-8 per group). (**G**) Representative H&E staining of eWAT from the first cohort (Figure 7B-D and supplemental figure 7B) of HFD mice treated with vehicle or ESI09 for one week (corresponding to Figure 7B-D). Arrows indicate immune cell infiltration and crown like structures (n=4 mice per group). (**A**) was analyzed with 2-way (sex x weight) ANOVA with Sidak posthoc. (**C**) Was analyzed by independent samples ttest. (**D-E**) data were analyzed by two-way mixed model ANOVAs (drug x time) with Fisher's LSD posthoc comparisons. (**F**) was analyzed with 2-way (phase x drug) ANOVA with Sidak posthoc. * equals significance from control unless otherwise specified. All error bars are standard error of the means.

Figure s8

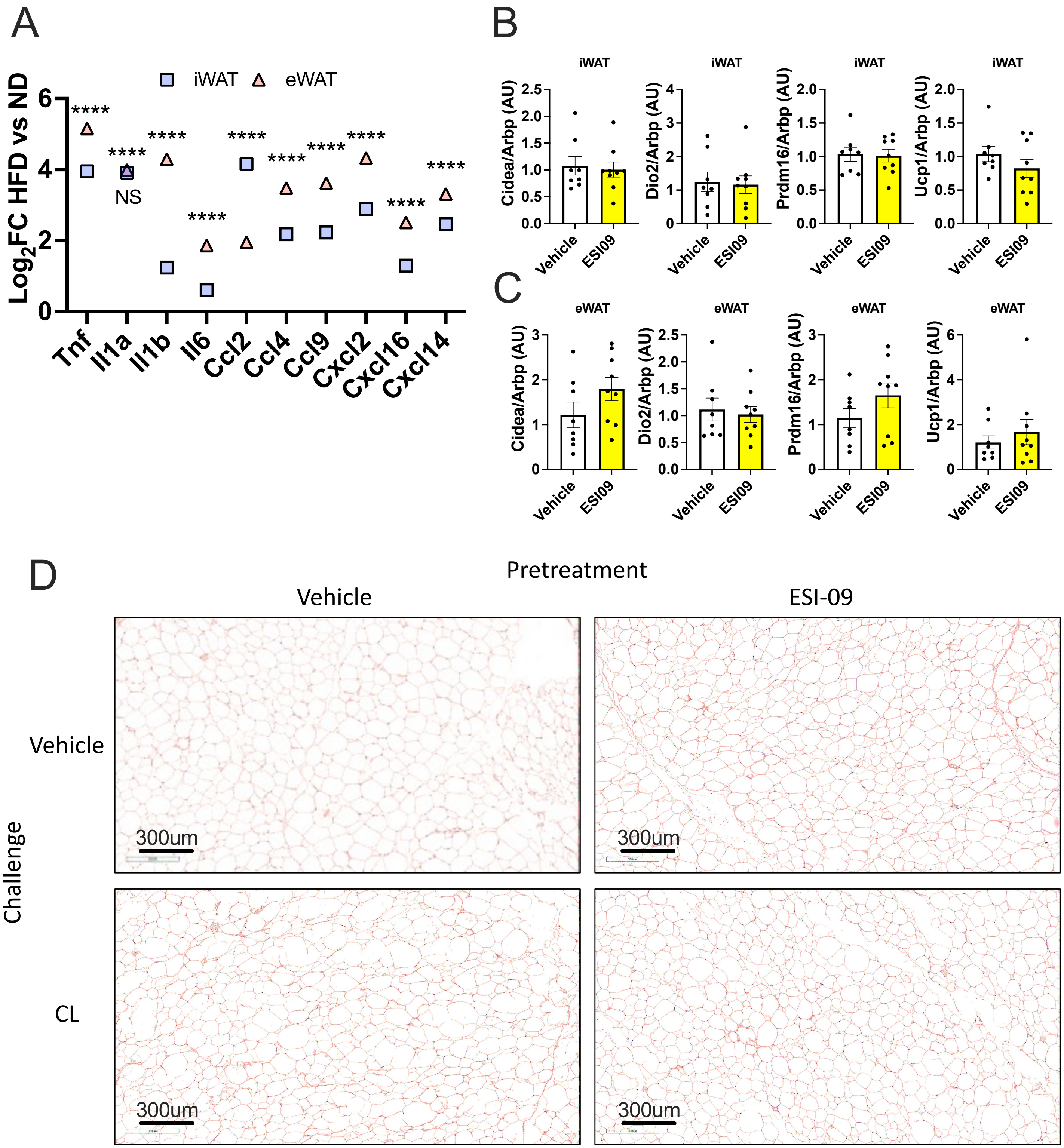
A
iWAT
eWAT
Log2FC HFD vs ND
****
NS
Tnf
Il1a
Il1b
Il6
Ccl2
Ccl4
Ccl9
Cxcl2
Cxcl16
Cxcl14
B
iWAT
Cidea/Arbp (AU)
Dio2/Arbp (AU)
Prdm16/Arbp (AU)
Ucp1/Arbp (AU)
Vehicle
ESI09
C
eWAT
D
Pretreatment
Vehicle
ESI-09
Challenge
CL
300um

Figure S8: (**A**) Assessment of inflammatory gene transcription using RNAseq on isolated adipocytes from iWAT and eWAT of mice on a HFD for 16 weeks (n=3 per group). (**B-C**) qPCR of browning genes from iWAT and eWAT respectively of mice (corresponding to Figure 7B-D and supplemental figure 7B); 24hrs after ESI09 treatment cessation mice were injected with CL-316243 0.1mg/kg or saline for 20mins and tissues harvested (n=8-9 per group). (**D**) UCP1 staining of iWAT from mice (corresponding to Figure 7B-C); 24hrs after ESI09 treatment cessation mice were injected with CL-316243 0.1mg/kg or saline for 20mins and tissues harvested (n=4 per group). Data in (**A**) were calculated with t-tests and false discovery rates applied to correct for multiple comparisons and (**B-C**) were analyzed by independent samples t-tests. * equals significance from control unless otherwise specified. All error bars are standard error of the means.

Figure s9

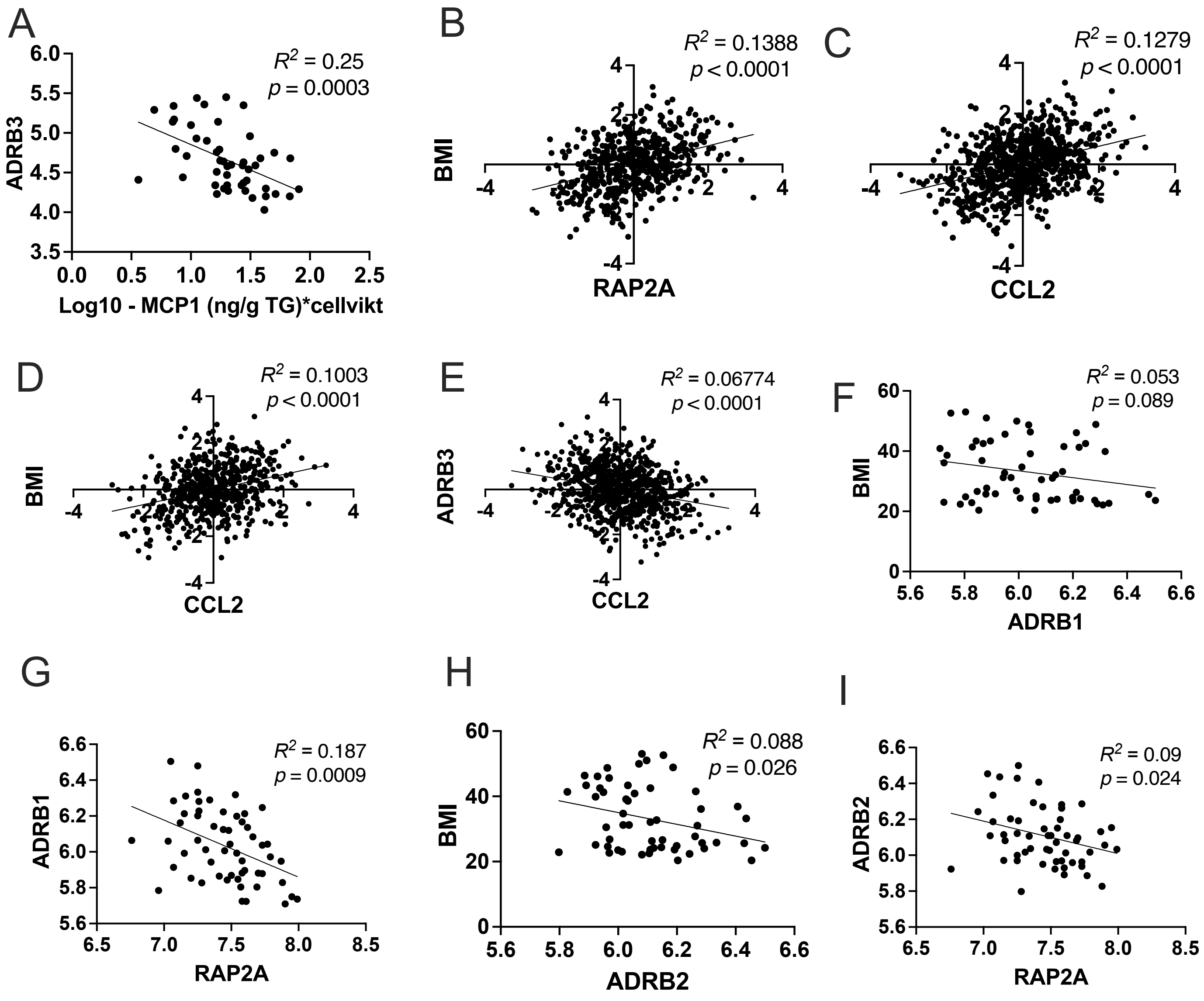
A
R^2 = 0.25
p = 0.0003
ADRB3
Log10 - MCP1 (ng/g TG)*cellvikt
B
R^2 = 0.1388
p < 0.0001
BMI
RAP2A
C
R^2 = 0.1279
p < 0.0001
CCL2
D
R^2 = 0.1003
p < 0.0001
BMI
CCL2
E
R^2 = 0.06774
p < 0.0001
ADRB3
CCL2
F
R^2 = 0.053
p = 0.089
BMI
ADRB1
G
R^2 = 0.187
p = 0.0009
ADRB1
RAP2A
H
R^2 = 0.088
p = 0.026
BMI
ADRB2
I
R^2 = 0.09
p = 0.024
ADRB2
RAP2A

Figure S9: (**A**) Correlations between isolated adipocyte secretion of MCP-1 and gene expression from microarray of human abdominal subcutaneous adipose tissue from women with varying BMI with anthropometric measurements (n=56). (**B-E**) Correlations between gene expression from RNAseq of human abdominal subcutaneous adipose tissue from men with varying BMI with anthropometric measurements (n=770). (**F-I**) Gene-gene and gene-anthropometric measurement regressions were performed on gene expression from microarray of human abdominal subcutaneous adipose tissue from women with varying BMI with anthropometric measurements (n=56). * equals significance from control unless otherwise specified. (**A-I**) were analyzed by linear-regression.

Figure S10

A

Correlation Matrix

	BMI	RAP2A	ADRB3	TRIB1	TNF	CCL2
BMI	1	0.558	-0.371	0.115	0.099	0.414
RAP2A	0.558	1	-0.432	-0.016	-0.182	0.44
ADRB3	-0.371	-0.432	1	-0.065	0.122	-0.34
TRIB1	0.115	-0.016	-0.065	1	0.472	0.708
TNF	0.099	-0.182	0.122	0.472	1	0.234
CCL2	0.414	0.44	-0.34	0.708	0.234	1

B

Model Results

Model Summary

Model	R	R Square	Adjusted R Square	Std. Error of the Estimate	Change Statistics				
					R Square Change	F Change	df1	df2	Sig. F Change
1	.627a	0.394	0.333	8.07609	0.394	6.489	5	50	<.001

a Predictors: (Constant), CCL2, TNF, ADRB3, RAP2A, TRIB1

Coefficients a

Model		Unstandardized Coefficients		Standardized Coefficients	t	Sig.	95.0% Confidence Interval for B
		B	Std. Error	Beta			Lower Bound
1	(Constant)	-108.041	52.553		-2.056	0.045	-213.597
	RAP2A	15.656	5.425	0.428	2.886	0.006	4.76
	ADRB3	-3.217	2.854	-0.142	-1.127	0.265	-8.949
	TRIB1	-2.872	3.561	-0.157	-0.806	0.424	-10.025
	TNF	8.76	5.29	0.212	1.656	0.104	-1.866
	CCL2	2.487	2.155	0.239	1.154	0.254	-1.841

a Dependent Variable: BMI

Figure S10: (**A**) Correlation matrix and (**B**) model results from multiple regression with BMI as the constant and gene expression data from microarray of human abdominal subcutaneous adipose tissue from women with varying BMI with anthropometric measurements as predictors (n=56).
